# Unbiased Single‐Cell Sequencing of Hematopoietic and Immune Cells from Aplastic Anemia Reveals the Contributors of Hematopoiesis Failure and Dysfunctional Immune Regulation

**DOI:** 10.1002/advs.202304539

**Published:** 2023-12-25

**Authors:** Rongqun Guo, Jingjing Kong, Ping Tang, Shuya Wang, Lina Sang, Liu Liu, Rong Guo, Ketai Yan, Mochu Qi, Zhilei Bian, Yongping Song, Zhongxing Jiang, Yingmei Li

**Affiliations:** ^1^ Department of Hematology The First Affiliated Hospital of Zhengzhou University Zhengzhou Henan 450052 China; ^2^ Academy of Medical Science Henan Medical College of Zhengzhou University Zhengzhou Henan 450052 China; ^3^ Department of Blood Transfusion The First Affiliated Hospital of Zhengzhou University Zhengzhou Henan 450052 China

**Keywords:** aplastic anemia, ferroptosis, mass spectrometric analysis, oxidized fatty acids metabolome, scRNA‐seq

## Abstract

Aplastic anemia (AA) is a bone marrow (BM) failure syndrome mediated by hyperactivated T‐cells with heterogeneous pathogenic factors. The onset of BM failure cannot be accurately determined in humans; therefore, exact pathogenesis remains unclear. In this study, a cellular atlas and microenvironment interactions is established using unbiased single‐cell RNA‐seq, along with multi‐omics analyses (mass cytometry, cytokine profiling, and oxidized fatty acid metabolomics). A new KIR^+^CD8^+^ regulatory T cells (Treg) subset is identified in patients with AA that engages in immune homeostasis. Conventional CD4^+^ T‐cells differentiate into highly differentiated T helper cells with type 2 cytokines (IL‐4, IL‐6, and IL‐13), GM‐SCF, and IL‐1β. Immunosuppressive homeostasis is impaired by enhanced apoptosis of activated Treg cells. Pathological Vδ1 cells dominated the main fraction of γδ T‐cells. The B/plasma, erythroid, and myeloid lineages also exhibit substantial pathological features. Interactions between TNFSF12‐TNFRSF12A, TNF‐TNFRSF1A, and granzyme‐gasdermin are associated with the cell death of hematopoietic stem/progenitor (HSPCs), Treg, and early erythroid cells. Ferroptosis, a major driver of HSPCs destruction, is identified in patients with AA. Furthermore, a case of twins with AA is reported to enhance the persuasiveness of the analysis. These results collectively constitute the cellular atlas and microenvironment interactions in patients with AA and provide novel insights into the development of new therapeutic opportunities.

## Graphical abstract

Aplastic anemia (AA) is a bone marrow failure syndrome mediated by hyperactivated T‐cells with heterogeneous pathogenic factors. A cellular atlas and microenvironment interactions is established using unbiased single‐cell RNA sequencing (scRNA‐seq) with the help of multi‐omics analysis (mass spectrometric analysis, cytokine profile, and oxidized fatty acids metabolome), and provide novel insights into the development of new therapeutic opportunities.

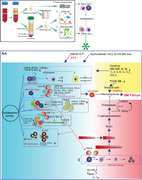

## 1 Introduction

Aplastic anemia (AA) is a bone marrow failure (BMF) disease characterized by pancytopenia. Considerable evidence has indicated that patients with AA exhibit hyperactive T‐cell responses.^[^ ^1^ ^]^ Single‐cell RNA sequencing (scRNA‐seq) analyses confirmed abnormal T‐cell states in clinical samples.^[^ ^2^ ^]^ However, data from patients and animal models have suggested the existence of additional dysfunctional immune cell types.^[^ ^3^ ^]^ Therefore, an in‐depth investigation of the unbiased immune and hematopoietic cell types in vitro is required.

Interferon (IFN)‐γ, IFN‐α, and IFN‐β exposure may deplete HSPCs or indirectly impair hematopoietic homeostasis through microenvironmental niche cells.^[^ ^4^ ^]^ Tumor necrosis factor‐alpha (TNF‐α) is a well‐known negative regulator of hematopoiesis, but its roles in AA are controversial.^[^ ^5^ ^]^ Lymphocyte infusion models in mice mirror many human observations; however, this type of AA mouse model is a graft‐versus‐host disease (GvHD) model. Activated CD8^+^ T, pathological TH17, TH1, and defective regulatory T cells (Treg)/regulatory B (Breg) cells contribute to immune dysregulation in acquired AA.^[^ ^6^, ^7^ ^]^ Natural killer (NK) cells have been identified as inhibitors of autologous CD8^+^ T‐cells, depending on the NKG2D (KLRK1)‐MICA axis, which is related to the disease state.^[^ ^8^ ^]^ γδ T‐cells can inhibit erythroid development in patients with acquired pure red cell AA.^[^ ^9^ ^]^ Although scRNA‐seq datasets of T‐cells, HSPCs, and NK cells have been provided,^[^ ^8^ ^]^ the AA comprehensive and unbiased immune landscape remains unclear.

Here, we revealed the panoramic landscape of AA using multi‐omics (scRNA‐seq, mass cytometry (MC), cytokine profiling, and oxidized fatty acid (OFAs) metabolomics). We found that GM‐CSF, IL‐1β, IL‐4, IL‐6, CCL2, and IL‐13 levels were upregulated in the BM supernatant. Th0 cells are forced into a highly differentiated state, and CD4^+^ Treg‐mediated immune homeostasis is impaired with increased apoptotic activity through the FAS‐CASP8 axis. AA CD8^+^ T cells showed increased cytotoxicity and induced the apoptosis of target cells through FASLG. We also identified a unique CD8^+^ Treg subset with NK properties, and found the δ1/δ2 T cell skew was reversed. B cell lineages are important participants in the hyperactive immune response. Using ligand‐receptor (L‐R) interaction analyses, we found that missing erythropoietin receptor (EPOR)^high^ early erythroid progenitors regulated immune homeostasis by providing abundant ligands, such as BAG6‐NCR3‐PS and NECTIN1‐CD96; however, late erythroid progenitors did not. Myeloid differentiation of AA is likely a result of GM‐CSF and IL‐4 driving pro‐inflammatory patterns. Dysregulation of iron homeostasis drives ferroptosis in HSPCs. Furthermore, the case of twins with AA presented with clinical features, scRNA‐seq, and MC analysis, which confirmed our results.

## 2 Results

### 2.1 Unbiased Single‐Cell Transcriptomic Landscape, Mass Cytometry Analyses, and Cytokine Profiling of AA

**Figure 1:**
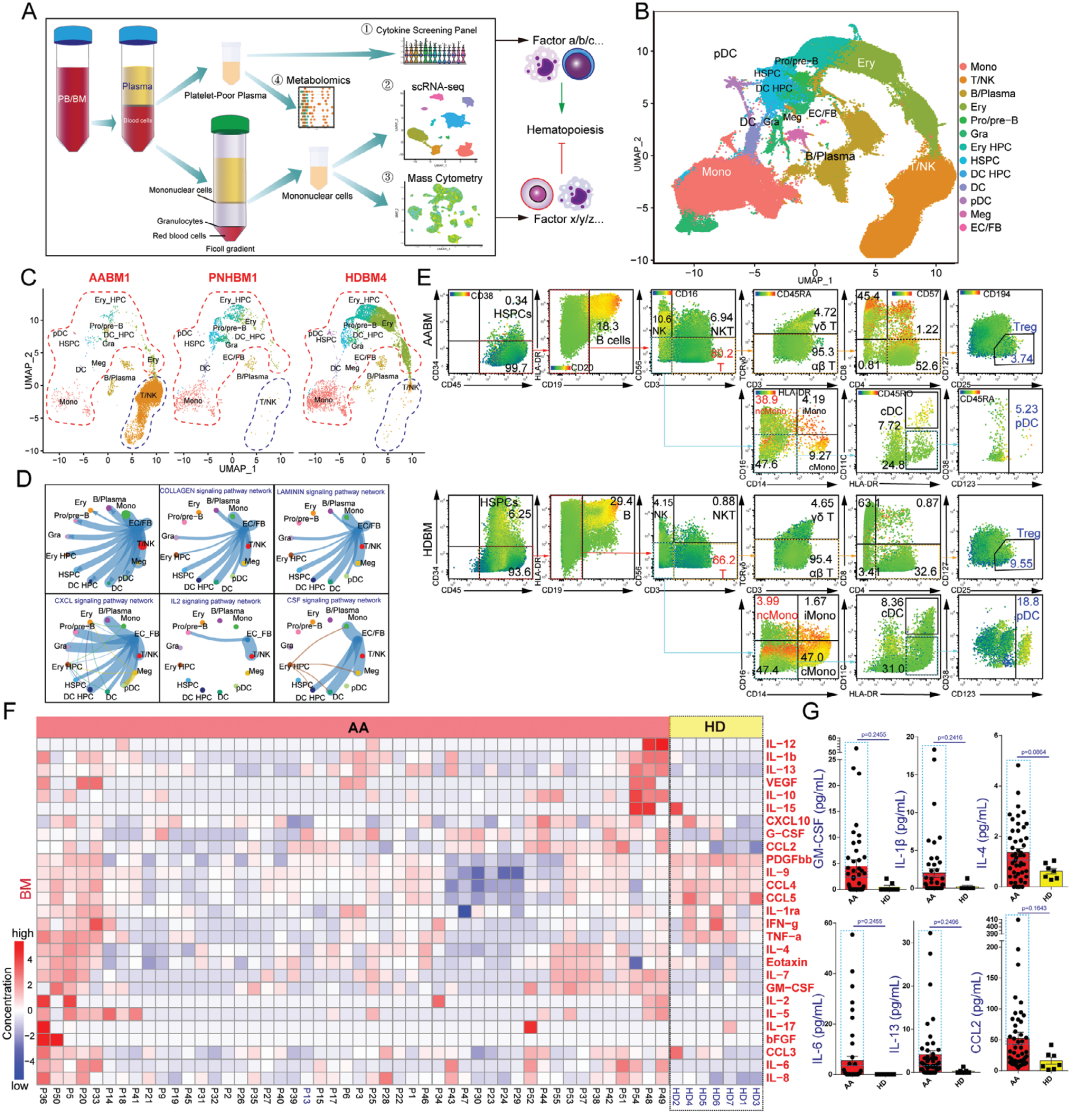
Dissecting hematopoietic cells and cytokine profile of patients with AA and HDs.A) Schematic of the study design. B) Uniform Manifold Approximation and Projection (UMAP) of mononuclear cells (MNCs) from BM and PB of HDs and patients with AA or PNH, colored by cell type. Mono: Monocytes; T/NK: T‐cells and NK cells; B/Plasma: B cells and plasma cells; Ery: erythroid cells; Pro/pre‐B: pro‐B and pre‐B cells; Gra: granulocytes; Ery HPC: erythroid progenitor cells; HSPCs: hematopoietic stem/progenitor cells; DC HPC: dendritic cell‐biased precursors; DC: dendritic cells; pDC: plasmacytoid dendritic cells; Meg: megakaryocyte‐biased cells; EC/FB: endothelial cells and fibroblasts. C) UMAP of BMNCs from typical BM samples of HDs and patients with AA or PNH, colored by cell type. Red dashed lines indicate cells other than B, plasma, and T/NK cells. The blue dashed lines indicate T/NK clusters. D) Circle plot showing cellular communication of non‐hematopoietic stromal cells and hematopoietic cells. The thickness of the flow indicates the contribution of the cell group or signaling pathway to each latent pattern. Several representative signaling pathway networks from stromal cells are shown. E) Representative dot plots of BM subset repartitioning in an HD and a patient with AA, were analyzed by mass cytometry. Dead and granulocyte‐like cells were removed. cMono: classical monocytes. Dot plot coloring is based on the expression level of the target surface marker, blue indicates the lowest expression, and red indicates the highest expression. F) Cytokine profiles of BM plasma samples. Heatmap showing the relative cytokine concentrations in the BM plasma from patients with AA (n = 48) and HDs (n = 7). G) Column charts presented the concentration of representative cytokines (GM‐CSF, IL‐1β, IL‐4, IL‐6, IL‐13, and CCL2). *P* value was determined by unpaired two‐tailed Student's *t*‐test: ^*^ *p* < 0.05, ^**^ *p* < 0.01, ^***^ *p* < 0.001, ^****^ *p* < 0.0001.

We used scRNA‐seq analysis, a cytokine screening panel, MC analysis, and metabolomics to establish a multi‐omics profile to distinguish the BM niche and immune components (**Figure** **1**A). We merged 13 samples from nine patients with AA, four samples from three patients with paroxysmal nocturnal hemoglobinuria (PNH), and eight samples from four healthy donors (HDs). Approximately 14 clusters were identified (Figure 1B; Figure S1A, Supporting Information), that supported a phenotype of T cell accumulation in the BM niche of patients with AA (Figure 1C; Figure S1B, Supporting Information). Additionally, BM mononuclear cells (BMMCs) of patients with PNH did not contain excessive T‐cells. L‐R interaction analysis showed that stromal cell populations (endothelial cells and fibroblasts) are critical contributors to cell‐cell communication (Figure 1D; Figure S1C, Supporting Information), which maintains hematopoiesis or immune homeostasis mediated by collagens, laminins, CXCL chemokines, IL‐7, and CSF family cytokines. We also identified the cell fraction of peripheral blood mononuclear cells (PBMCs) or BMMCs from HDs and patients with AA using MC analysis (Figure 1E; Figure S1D, and Table S2, Supporting Information). Compared with HDs, the fractions of Tregs and plasmacytoid dendritic cells (pDCs) in AA BM were lower. The fractions of T‐cells, non‐classical monocytes (ncMono), and intermediate monocytes (iMono) increased, which is consistent with the hyperactivated immune response in patients with AA.

Cytokine mapping of the BM plasma showed that the concentrations of IL‐9, CCR5 ligands (CCL3/4/5), and PDGF‐BB were higher in the HD BM plasma than that in the AA BM plasma (Figure 1F; Figure S1E, Supporting Information). Part of AA samples had higher concentrations of GM‐CSF, IL‐1β, IL‐4, IL‐6, IL‐13, and CCL2 than those in HD BM plasma (Figure 1G). TNF‐α and IFN‐γ were not significantly higher in BM of patients with AA compared with those in HD BM (Figure S1E, Supporting Information). These biological differences may explain the paradoxical results observed at different stages of disease progression; thus, anti‐TNF therapies cannot become universal AA treatment.^[^ ^10^ ^]^

### 2.2 Hyperactivation of Cytotoxic CD8^+^ T‐cells Potentially Triggers Cell Death of HSPCs via Apoptosis and Pyroptosis

**Figure 2:**
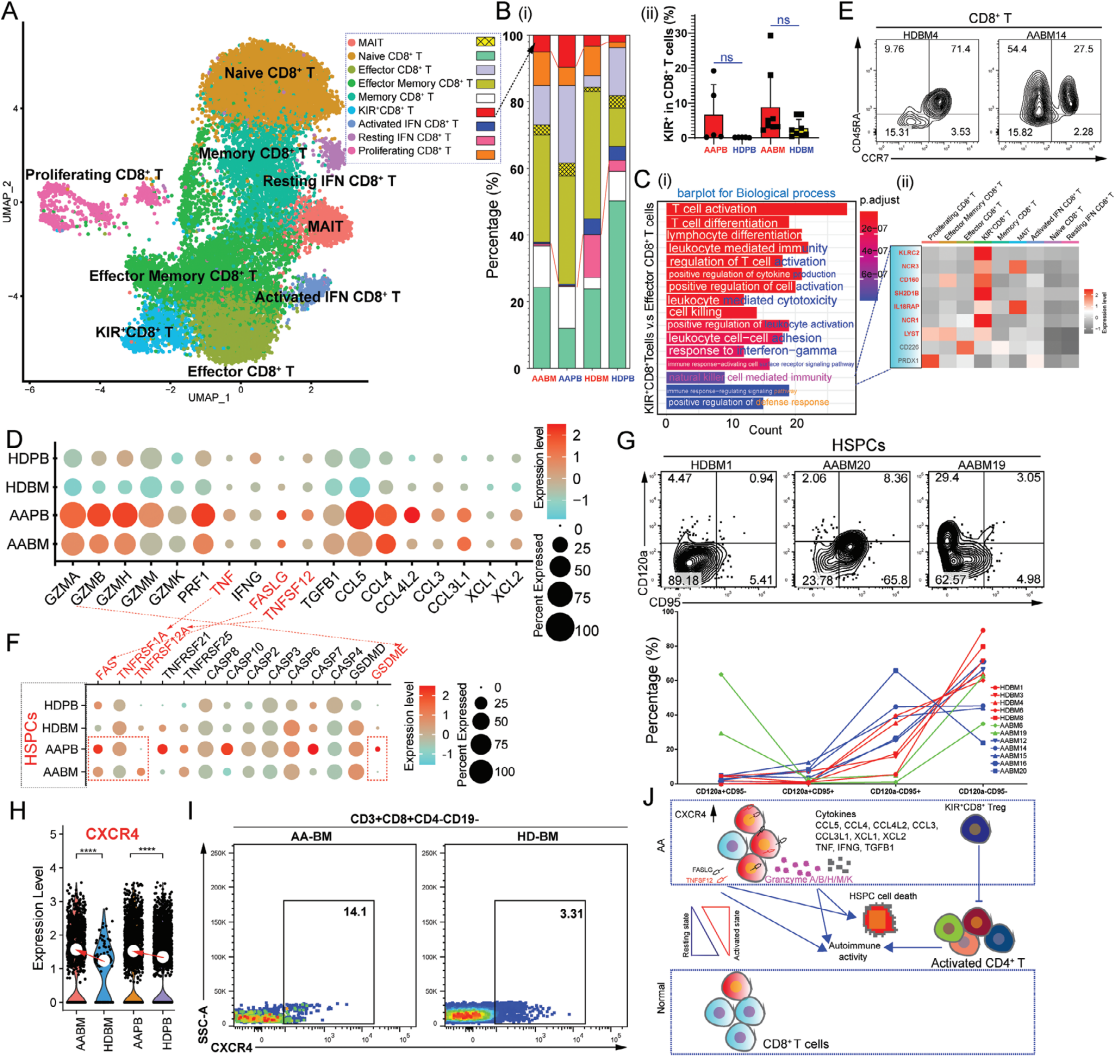
Characterizing CD8^+^ T subpopulations and their effectors in HDs and patients with AA. A) UMAP plot of CD8^+^ T‐cells. Bi) The bar plot shows the proportion of each cell cluster in each sample. Resting CD8^+^ T‐cells include naïve CD8^+^ T‐cells, memory CD8^+^ T‐cells, and IFN‐resting CD8^+^ T‐cells. ii) Percentage (%) of cells expressing KIR (KIR2DL1, KIR2DL2/DL3, KIR2DL5, KIR3DL1, and KIR3DL2) in CD8^+^ T‐cells, detected using flow cytometry. C) GO analysis of biological processes based on the DEGs of KIR^+^CD8^+^ T‐cells and CD8^+^ effectors i). Gene lists of “T cell differentiation” and “lymphocyte mediated immunity” were duplicates, so we only retained the former. Gene lists of “lymphocyte differentiation” and “mononuclear cell differentiation” were duplicates, and only the former was retained. Gene lists of “immune response‐activating cell surface receptor signaling pathway,” “immune response‐activating signal transduction,” and “immune response‐regulating cell surface receptor signaling pathway” were duplicates; only the former was retained. The heatmap shows the average expression of genes from the “natural killer cell mediated immunity” geneset ii). D–F) Dot plots show the expression of indicated genes in CD8^+^ T‐cells of each group (D) and HSPCs of each group (F). Red arrows and letters indicate ligand‐receptor pairs.(E) MC analysis of BM CD8^+^ T‐cells from HD and patient with AA. G) MC analysis of BM HSPCs from HD and patients with AA. H) Violin plots show *CXCR4* expression in CD8^+^ T‐cells of different groups. I) Representative flow cytometry plots showing CXCR4 expression in BM CD8^+^ T‐cells from patients with AA and healthy donors. CD8^+^ T‐cells were defined as CD19^−^TCRγδ^−^CD3^+^CD4^−^CD8^+^ cells. J) Schematic of the proposed CD8^+^ T‐mediated autoimmune response and HSPCs destruction process.

Reanalysis of subtle transcriptional shifts is essential for distinguishing different subpopulations.^[^ ^11^ ^]^ The CD8^+^ T‐cells were divided into nine subsets (**Figure** **2**A). We found a decreased frequency of resting CD8^+^ T‐cells in the PBMCs or BMMCs of patients with AA compared to that in HDs (Figure 2B). Killer cell immunoglobulin‐like receptors (KIRs)‐expressing CD8^+^ (KIR^+^CD8^+^) T‐cells have been identified as novel regulatory CD8^+^ T‐cells that can suppress self‐reactive or otherwise pathogenic cells without affecting immune responses against pathogens.^[^ ^12^ ^]^ We also found this CD8^+^ T subset (Figure S2A, Supporting Information) and identified that the frequency of KIR^+^CD8^+^ T‐cells was significantly higher in patients with AA than that in HDs (Figure 2Bi). This tendency of increasing KIT^+^CD8^+^ T‐cells was also confirmed by flow cytometry (Figure 2Bii; Figure S2B, Supporting Information). KIR^+^CD8^+^ T‐cells showed cytotoxic features similar to those of other CD8^+^ effector T‐cells (Figure S2C, Supporting Information). Gene Ontology (GO) analysis revealed that some differential genes were enriched in the biological processes of “natural killer cell‐mediated immunity” (Figure 2C). Compared with CD8^+^ T‐cells from HDs, CD8^+^ T‐cells from patients with AA expressed higher levels of cytotoxic and cytokine genes (Figure 2D). MC analysis also identified that AA CD8^+^ T‐cells mainly comprised effector cells (Figure 2E; Figure S2D, Supporting Information). FAS was upregulated in HSPCs of patients with AA (Figure 2F). The upregulation of *TNF* and *TNFSF12* in AA CD8^+^ T‐cells suggests that the TNF‐TNFR1 axis‐mediated cell death is involved in the destruction of HSPCs. MC analysis revealed that CD120a (TNFR1) and FAS were upregulated in the BM CD34^+^ HSPCs of the patients (Figure 2G; Figure S2J, Supporting Information). The upregulation of *GSDME* in AA HSPCs showed that activated CD8^+^ T‐cells secreted granzyme B and perforin, which triggered pyroptosis in HSPCs.^[^ ^13^ ^]^ As a downstream target of IFN signaling, *TNFSF10* was decreased in the CD8^+^ T‐cells of patients with AA (Figure S2E, Supporting Information). Consistent with this, IFN‐resting/activated CD8^+^ T‐cells were the major source of *TNFSF10* (Figure S2F, Supporting Information), indicating that defective IFN signaling may be involved in AA pathology. Meanwhile, the expression patterns of “decoy” receptors and death domain‐containing receptors indicate that hyperactivated CD8^+^ T‐cells may not induce cell death of HSPCs via TNFSF10‐triggered signal, as previously reported^[^ ^14^ ^]^ (Figure S2G, Supporting Information).

BM CD8^+^ T‐cells from patients with AA expressed higher *CXCR4* than those from HDs (Figure 2H), as determined using flow cytometry at the protein level (Figure 2I). Notably, AA PB CD8^+^ T‐cells upregulated *CXCR4* mRNA but not protein level (Figure S2H, Supporting Information). In addition, *CD69* expression was upregulated in AA CD8^+^ T‐cells (Figure S2I, Supporting Information). These results indicate that CD8^+^ T cell migration is dysfunctional in patients with AA. Altogether, we described CD8^+^ regulatory T‐cells and a novel mode of CD8^+^ T cell‐mediated destruction of HSPCs and Tregs in patients with AA (Figure 2J; Figure S2J, Supporting Information).

### 2.3 The Activated TH2‐State of Conventional CD4^+^ T‐cells is Primed, and the Suppressive Capacity of Tregs is Impaired by Increasing Sensitivity of Cell Death

**Figure 3:**
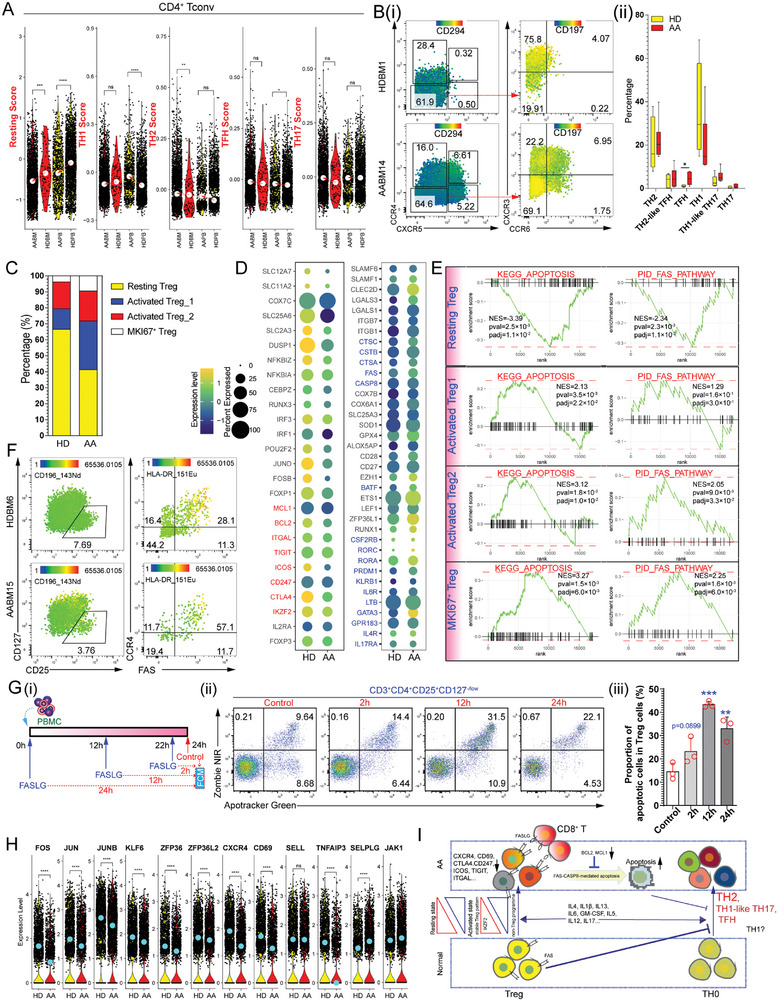
Characterization of CD4^+^ Tconv cells and Treg in patients with AA. A) Violin plots of resting, TH1 (type 1 T helper), TH2 (type 2 T helper), TFH (T follicular helper), and TH17 (type 17 T helper) scores in CD4^+^ Tconv cells. B) Representative CCR4, CXCR5, CXCR3, and CCR6 in BM non‐Treg CD4^+^ T‐cells of HD and patient with AA i). Percentage of T helper subsets (TH2: CCR4^+^CXCR5^−^, TH2‐like TFH: CCR4^+^CXCR5^+^, TFH: CCR4^−^CXCR5^+^, TH1: CCR4^−^CXCR5^−^CXCR3^+^CCR6^−^, TH1‐like TH17: CCR4^−^CXCR5^−^CXCR3^+^CCR6^+^, TH17: CCR4^−^CXCR5^−^CXCR3^−^CCR6^+^) ii). C) The bar plot shows the proportion of major Treg subpopulations in each sample group. D) The dot plot shows the genes specifically expressed in primary CD8^+^ T‐cells in different groups. Yellow represents high expression, and dark green represents low expression. The circle size represents the percentage of cells expressing the indicated genes. E) The gene set enrichment analysis (GSEA) enrichment score revealed the difference of pathways (KEGG_APOPTOSIS and PID_FAS_PATHWAY) in major Treg subsets. F) Representative mass cytometry analysis of the surface markers CCR4 and FAS in Tregs from HDs and patients with AA. G) In vitro experimental setup i). Representative flow cytometry plots of apoptotic Treg after 2, 12, and 24 h under FASLG supplementing conditions ii). Bar plots of proportions of apoptotic Treg cells in the total Treg cells. H) Violin plots show specifically expressed genes (*FOS*, *JUN*, *JUNB*, *KLF6*, *ZFP36*, *ZFP36L2*, *CXCR4*, *CD69*, *SELL*, *TNFAIP3*, *SELPLG*, and *JAK1*) in Treg cells of different groups. I) Schematic of the proposed process of the loss of CD4^+^ Tconv cell homeostasis and impairment of Tregs.

We extracted the data matrix of the CD4^+^ T cluster from the scRNA‐seq datasets of patients with AA and PNH, and HDs. Then, these CD4^+^ T‐cells were reanalyzed and Uniform Manifold Approximation and Projection (UMAP) was performed (Figure S3A, Supporting Information). Differential gene expression from the scRNA‐seq data resolved CD4^+^ T‐cells into five clusters (Figure S3B, Supporting Information). We used several gene sets to assess the direction of T helper cell (TH) polarization (Table S5, Supporting Information). The resting score of conventional CD4^+^ T (CD4^+^ Tconv) cells decreased in patients with AA and activated markers (*IL2RA* and *TNF*) were upregulated (Figure S3C, Supporting Information). We also investigated whether differentiation and polarization of the resting states into TH1/TH2/TFH states were enhanced (**Figure** **3**A). MC analysis showed that BM TFH and TH1‐like TH17 cells could be regarded as pathological immune cells (Figure 3B). *TNFSF10* expression was downregulated in CD4^+^ Tconv cells (Figure S3C, Supporting Information). Cytokine profile analysis has shown that the upregulation of IL‐4/13 in the BM plasma, which robustly supports the type 2 inflammation‐mediated TH2 pattern, is a hallmark of AA.

We sorted Treg cells from the BM of one HD for scRNA‐seq assay and merged this scRNA‐seq dataset with Tregs from patients with AA (Figure S3D, Supporting Information). Treg cells were classified into four subsets (Figure S3E, Supporting Information). The resting Treg subset expressed naïve/memory features but lacked effector and activated features (Figure S3F, Supporting Information). Resting/activated states were identified using gene set enrichment analysis (GSEA) based on two Treg‐related genesets (GSE15659_RESTING_VS_ACTIVATED_TREG_DN and GSE15659_RESTING_VS_ACTIVATED_TREG_UP) (Figure S3G, Supporting Information). Compared with Tregs from HD BM, the resting Treg subset was decreased in patients with AA (Figure 3C). The TH2 and TH17 signatures were increased in the Tregs of patients with AA, which was assessed by the upregulation of *GATA3*, *LTB*, *IL6R*, and *PRDM1* (Figure S3H, Supporting Information). Compared with Tregs in HD BM, Tregs from patients with AA expressed immune‐suppressive molecules (*ITGAL*, *ICOS*, *CD247*, and *CTLA4*) at low levels. AA Tregs expressed high levels of apoptosis‐related genes and low levels of anti‐apoptosis‐related genes at low levels (Figure 3D). Several cell‐death‐related genesets have been used to assess apoptotic signaling in various Treg subsets. The resting Treg subset showed low levels of apoptotic signals, and the other three activated Treg subsets showed high levels of cell death signals (apoptosis, pyroptosis, and ferroptosis) (Figure 3E and Figure S3I, Supporting Information), suggesting that the death of AA Tregs was easily triggered when sufficient extrinsic and intrinsic signals were present (Figure 3F and Figure S3J, Supporting Information). To identify FAS‐mediated apoptosis of Treg, we cultured PBMC with FASLG protein supplementation (Figure 3Gi). As shown in Figure 3Gii, the proportion of apoptotic Tregs increased after FASLG protein treatment. Notably, cytotoxic cells‐derived FASLG contributed to the apoptosis of Treg cells (Figure 3Giii), indicating that the interruption of FAS‐mediated apoptosis is a potential target of AA.

AA Tregs expressed several important transcription factors (*FOS*, *JUN*, *JUNB*, *KLF6*, *ZFP36*, and *ZFP36L2*) at low levels, but *JAK1* expression was high. Transcripts of the migration genes *CXCR4* and *CD69* were downregulated in AA Tregs, whereas *SELPLG* was upregulated (Figure 3H), suggesting that the immunosuppressive function and migration of Tregs derived from patients with AA were impaired.

Collectively, our results demonstrate that patients with AA present impaired Tregs, leading to a hyperactivated T cell‐mediated autoimmune response. The activation of CD8^+^ T‐cells via FAS‐FASLG triggered Treg destruction with increased apoptotic sensitivity (Figure 3I).

### 2.4 Pathological Vδ1 T‐cells were Increased in Patients with AA

**Figure 4:**
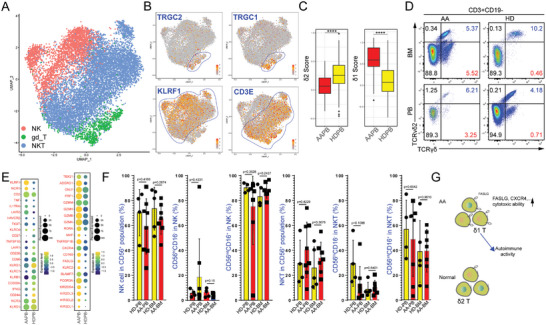
Dysfunctional γδ T‐cells in patients with AA. A) UMAP of scRNA‐seq data from the NK cells, NKT cells, and γδ T‐cells. γδ T: *CD3E* ^+^ *TRGC1* ^+^/*TRGC2* ^+^; NK: *CD3E* ^−^ *KLRF1* ^+^ *NCAM1* ^+^/*FCGR3A* ^+^; NKT: *CD3E* ^+^ *KLRF1* ^+^ *NCAM1* ^+^/*FCGR3A* ^+^. B) UMAP plot of representative expression patterns of *TRGC2*, *TRGC1*, *KLRF1*, and *CD3E*. Blue lines mark clusters with high *TRGC2*‐expression, *TRGC1*‐expression, *KLRF1*‐expression, or *CD3E*‐expression, respectively. C) δ1 T signature scores and δ2 T signature scores for γδ T‐cells in HDs and patients with AA. D) Representative flow cytometry plots of TCRvδ2^+^ T‐cells in CD3^+^CD19^−^ T‐cells from PB and BM samples of patients with AA and healthy donors. E) Dot plots exhibited the selected gene expression in γδ T‐cells of HDs and patients with AA. F) The percentage of different cell subtypes in CD56^+^ cells, NK cells, and NKT cells. G) Schematic of the proposed process of loss of δ1/δ2 homeostasis.

We extracted the data matrix of NK NKT, and γδ T‐cells by excluding CD4^+^ and CD8^+^ T‐cells from our scRNA‐seq datasets of patients with AA and PNH, and HDs. Three clusters were identified based on the expression levels of *TRGC2*, *TRGC1*, *KLRF1*, *CD3E*, *CD247*, *NCAM1*, *KLRB1*, *FCGR3A*, and *KLRC2* (**Figure** **4**A,B; Figure S4A, Supporting Information). The proportion of NK, NKT, and γδ T‐cells also changed in patients with AA, indicating these immune subsets are also involved in pathological states (Figure S4B, Supporting Information). Based on a previous study,^[^ ^15^ ^]^ we used two gene lists (δ1 gene list and δ2 gene list) for evaluating the δ1/δ2 skewing. The module scores showed δ1‐like T‐cells were dominant in the patients with AA instead of δ2‐like T‐cells (Figure 4C). We further analyzed the results using flow cytometry to identify the cell surface markers (Figure 4D). We observed a significant decrease in the TCRvδ2^+^ subset in γδ T‐cells (Figure S4C, Supporting Information). γδ T‐cells derived from patients with AA minimally expressed *KLRC1* (*NKG2A*) with high levels of *FASLG*, *TNF*, chemokines, and cytotoxic genes (Figure 4E), indicating the tendencies of cytotoxic activation. The MC analysis showed CD45RA^+^CD27^−^ effectors increased in AA BM γδ T‐cells (Figure S4D,E, Supporting Information). We also analyzed the fraction of NK and NKT subsets using MC analysis and found no obvious differences between the CD56^hi^CD16^−^ subset and CD56^int^CD16^+^ subset in the AA and HD groups (Figure 4F; Figure S4F, Supporting Information). Altogether, we distinguished the pathological states of γδ T‐cells, providing novel views on targeting these cell types (Figure 4G).

### 2.5 Increased Cell Death of B Lineages and Erythroid Progenitors, and Upregulated Pro‐Inflammatory Pattern of Myeloid Cells in Patients with AA

**Figure 5:**
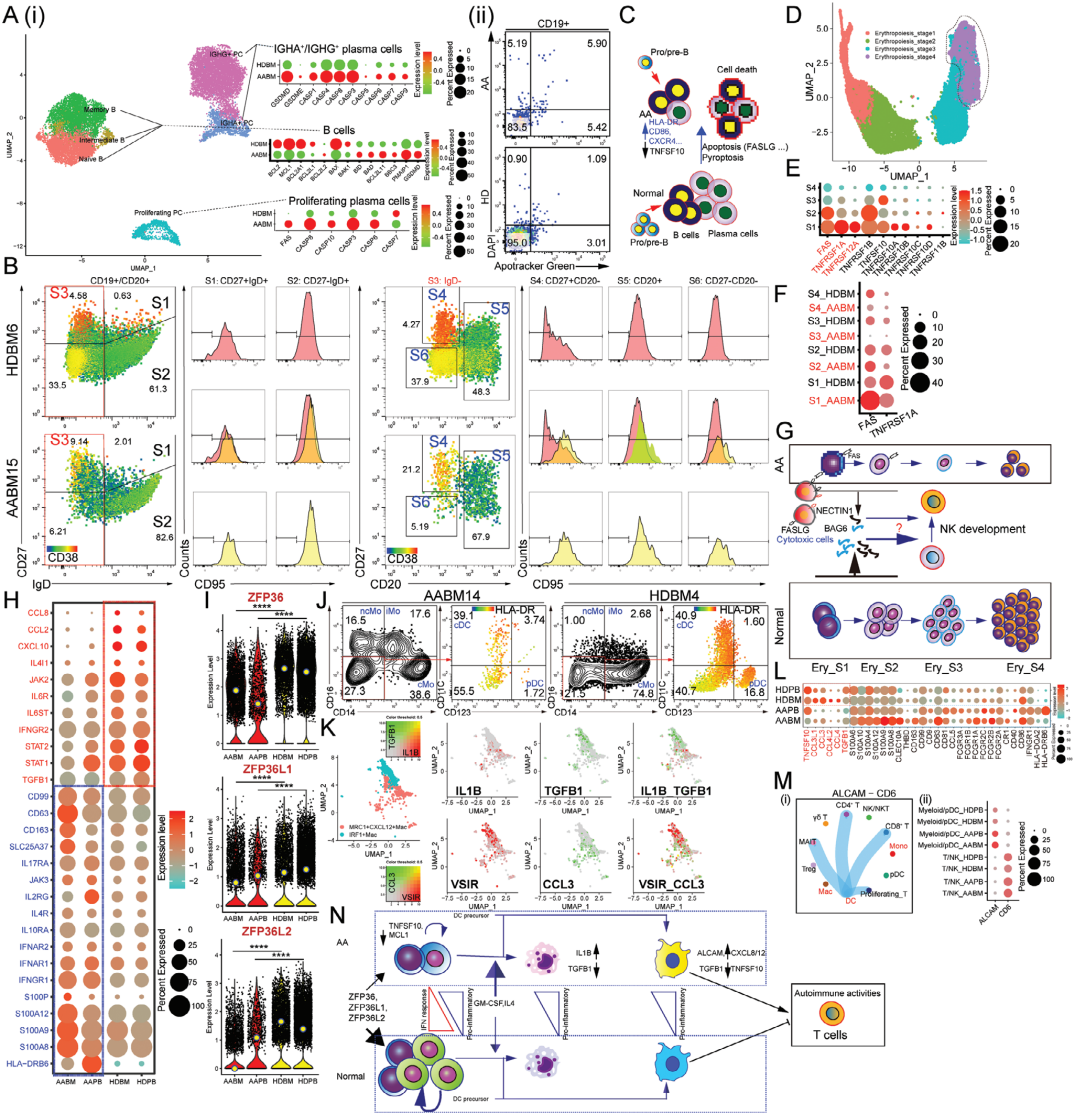
scRNA‐seq analysis highlighted pro‐inflammatory patterns of myeloid cells and increased cell death‐sensitive of B lineage subsets and erythroid progenitors. Ai) UMAP of the B lineages (B and plasma cells) profiled herein, with each cell color‐coded according to the cell cluster. Dot plots show cell death‐related genes in B cells, IGHA^+^/IGHG^+^ plasma cells, and proliferating plasma cells. ii) Flow cytometric analysis of apoptotic and dead CD19^+^ B cells. B) Representative plots of MC analysis showing the gating strategy of different B subsets (naïve B cells: CD27^−^IgD^+^, IgD^−^ memory B cells: IgD^−^CD20^+^, plasmablasts: IgD^−^CD20^−^CD27^+^), and histograms showing the expression of CD95. Dot plot coloring is based on CD38 expression; blue is the lowest expression and red is the highest expression. C) Schematic of the proposed process of cell death of B lineages in patients with AA. D) UMAP shows the distribution of erythroid differentiation stages. E) Dot plots exhibited the selected gene (*FAS*, *TNFRSF1A*, *TNFRSF12A*, *TNFRSF1B*, *TNFSF10*, *TNFRSF10A*, *TNFRSF10B*, *TNFRSF10C*, *TNFRSF10D*, and *TNFRSF11B*) expression in different erythroid stages. F) The dot plot exhibits the expression of FAS and TNFRSF1A in different stages, split by sample group. Dotplot coloring is based on the expression level of the target gene, grey is the lowest expression and red is the highest expression. G) Schematic of the proposed process of hyperactivated T cell‐mediated apoptosis of early erythroid cells and impaired NK cell development. H) The dot plot shows the scaled expression (color) and the percentage of non‐zero‐expressing cells (size) of selected genes per monocyte group. Red marks upregulated genes in HD samples, and blue marks downregulated genes. I) *ZFP* family (*ZFP36*, *ZFP36L1*, and *ZFP36L2*) expression depicted as violin plots across different groups. J) Representative MC gating in the BM myeloid lineages (cMo: CD14^+^CD16^−^, iMo: CD14^+^CD16^+^, ncMo: CD14^−^CD16^+^, cDC: CD14^−^CD16^−^CD11C^+^, pDC: CD14^−^CD16^−^CD11C^−^CD123^+^) of HDs and patients with AA. Dot plot coloring is based on HLA‐DR expression; blue is the lowest, and red is the highest. K) The expression levels of the selected genes were color‐coded and projected onto the UMAP of the macrophages. L) The dot plot shows the scaled expression (color) and percentage of non‐zero‐expressing cells (size) of the selected genes per group in DC. M) Identification of ALCAM‐CD6 as an important ligand‐receptor pair from DC to T‐cells i). The dot plot shows the expression levels of ALCAM and CD6 in different subsets and groups ii). Dotplot coloring is based on the expression level of the target gene; grey is the lowest, and red is the highest. N) Schematic of the proposed process of altered myeloid differentiation contributing to the autoimmune responses.

The data matrix of B cells and plasma cells (PCs) was extracted from our scRNA‐seq datasets, and these cells were reclustered into six subsets (**Figure** **5**Ai; Figure S5A, Supporting Information). AA‐derived BM B cells showed high apoptotic activity, with upregulated pro‐apoptotic genes (*BID*, *BAD*, *BCL2L11*, *BBC3*, and *PMAIP1*) and downregulated anti‐apoptotic genes (*BCL2*, *MCL1*, and *BCL2L1*). The expression of pyroptosis‐related genes (*GSDMD*, *GSDME*, *CASP1*, and *CASP4*) was increased in IGHA^+^/IGHG^+^ PCs. Proliferating PCs in the BM of patients with AA show a hallmark of FAS‐mediated apoptosis. MC analysis also identified the upregulation of FAS in the B cells of patients with AA as a notable characteristic. Increased cell death of B cell lineages in patients with AA was also identified using flow cytometry (Figure 5Aii; Figure S5B, Supporting Information). Compared with other B cell subsets, plasmablasts were more susceptible to FAS‐mediated apoptosis (Figure 5B), which was consistent with our scRNA‐seq analysis (Figure 5C). Furthermore, AA B cells may be involved in T cell‐mediated immunity via antigen presentation (Figure S5C,D, Supporting Information).

We divided the BM erythroid lineages into four stages (stage‐1 (S1, *MYC* ^high^ *MYB* ^high^; stage‐2 (S2), *EPOR* ^high^ *TFRC* ^high^; stage‐3 (S3), *SLC4A1* ^high^ *GYPA* ^high^; stage‐4 (S4), *IFIT1B* ^high^ *NCOA4* ^high^) (Figure 5D; Figure S5E, Supporting Information). The proportion of early stages (S1/2) would have decreased in BM erythropoiesis of patients with AA. The terminal stage (S4) showed a significant increase in the proportion of erythroid lineages and expressed *EPOR* at very low levels (Figure S5F, Supporting Information). Furthermore, early erythroid progenitors (S1/2) expressed *FAS*, *TNFRSF1A*, and *TNFRSF12A* at high levels (Figure 5E), indicating that early progenitors were more sensitive to extrinsic apoptotic protein ligands (*FASLG*, *TNF*, and *TNFSF12*) than late progenitors (S3/4). Compared with the early progenitors of HDs, those from patients with AA upregulated the expression of *FAS* (Figure 5F), suggesting that hyperactivated CD8^+^ T cell‐derived FASLG mediates the destruction of early progenitors with other ligands (TNF and TNFSF12). Notably, early progenitors (S1/2) showed a higher number and strength of interactions with T/NK cells than late progenitors (S3/4) (Figure S5G, Supporting Information). Early progenitors provided abundant immune regulation‐related ligands, such as *BAG6*, *NECTIN1*, *LGALS9*, *CDH1*, and *MIF*. BAG6‐NCR3 (NKp30), NECTIN1‐CD96, LGALS9‐CD44/CD45, CDH1‐KLRG1/ITGAE/ITGB7, and MIF‐CD74/CXCR4/CD44 signaling were involved in the development and function of NK cells ^[^ ^16^, ^17^ ^]^ (Figure S5H,I, Supporting Information). The disappearance of early erythroid progenitors via CD8^+^ T cell‐mediated cell death may impair NK cell development and homeostasis (Figure 5G). However, the mechanism of the erythroid‐NK interaction remains to be defined.

We examined the main compartments of myeloid cells and pDC in our dataset to identify the variability between the AA and HD groups (Figure S5J, Supporting Information). Three populations of cells were captured: monocyte/macrophages (Mono/Mac), dendritic cells (DCs), and neutrophils (Neu). We excluded residual Neu and compared the Mono/Mac and DC fractions (Figure S5K, Supporting Information). Monocytes from patients with AA showed reprogrammed cytokine‐driven immune responses with upregulated *IL17RA*, *JAK3*, *IL4R*, *IL2RG*, *IL10RA*, *IFNAR2*, *IFNAR1*, *IFNGR1*, and *S100* family members, but downregulated *CCL8*, *CCL2*, *CXCL10*, *IL6R*, *IL6ST*, *IFNGR2*, *STAT1*, *STAT2*, and *TGFB1* (Figure 5H). *ZFP36*‐family members were downregulated in AA monocytes (Figure 5I). MC analysis showed that the frequencies of ncMono and iMono were higher in the AA BM group than in the HD group (Figure 5J). The frequency of pDC in the BM of patients with AA is decreased, indicating that pDC may be involved in the pathological process of AA. Notably, *TNFSF10* expression was also reduced in monocytes of patients with AA (Figure S5L, Supporting Information). MRC1^+^CXCL12^+^ Mac mainly exists in the BM of patients with AA. Compared with IRF1^+^ Mac, this macrophage subpopulation showed an M1‐like pattern (*IL1B* and *CCL3*) (Figure 5K). The DCs of patients with AA were driven into a pro‐inflammatory state with high expression of *S100A* family members, *CD40*, *CD86*, and *IFNGR1*, indicating an active DC‐priming T‐cell response (Figure 5L). The interaction relationship between DC and other cell types showed that DC also provides abnormal immune signals via ALCAM‐CD6 and upregulate the expression levels of *CD6* in T/NK cell subsets and *ALCAM* in DC subsets of patients with AA (Figure 5M). DC‐derived LGALS9 and CD86 acted as immune checkpoints for dysfunctional immune responses (Figure S5M, Supporting Information). Our data revealed that myeloid differentiation was impaired by exposure to excessive GM‐CSF, IL‐4, and other cytokines and that the pro‐inflammatory patterns contributed to autoimmune activities via the downregulation of some key genes (ZFP36 families, TNFSF10, and TGFB1) and upregulation of ALCAM and cytokines (IL1B, CXCL8, and CXCL12) (Figure 5N).

### 2.6 Ferroptosis‐Driven Cell Death Contributed to Hematopoiesis Failure in Patients with AA

**Figure 6:**
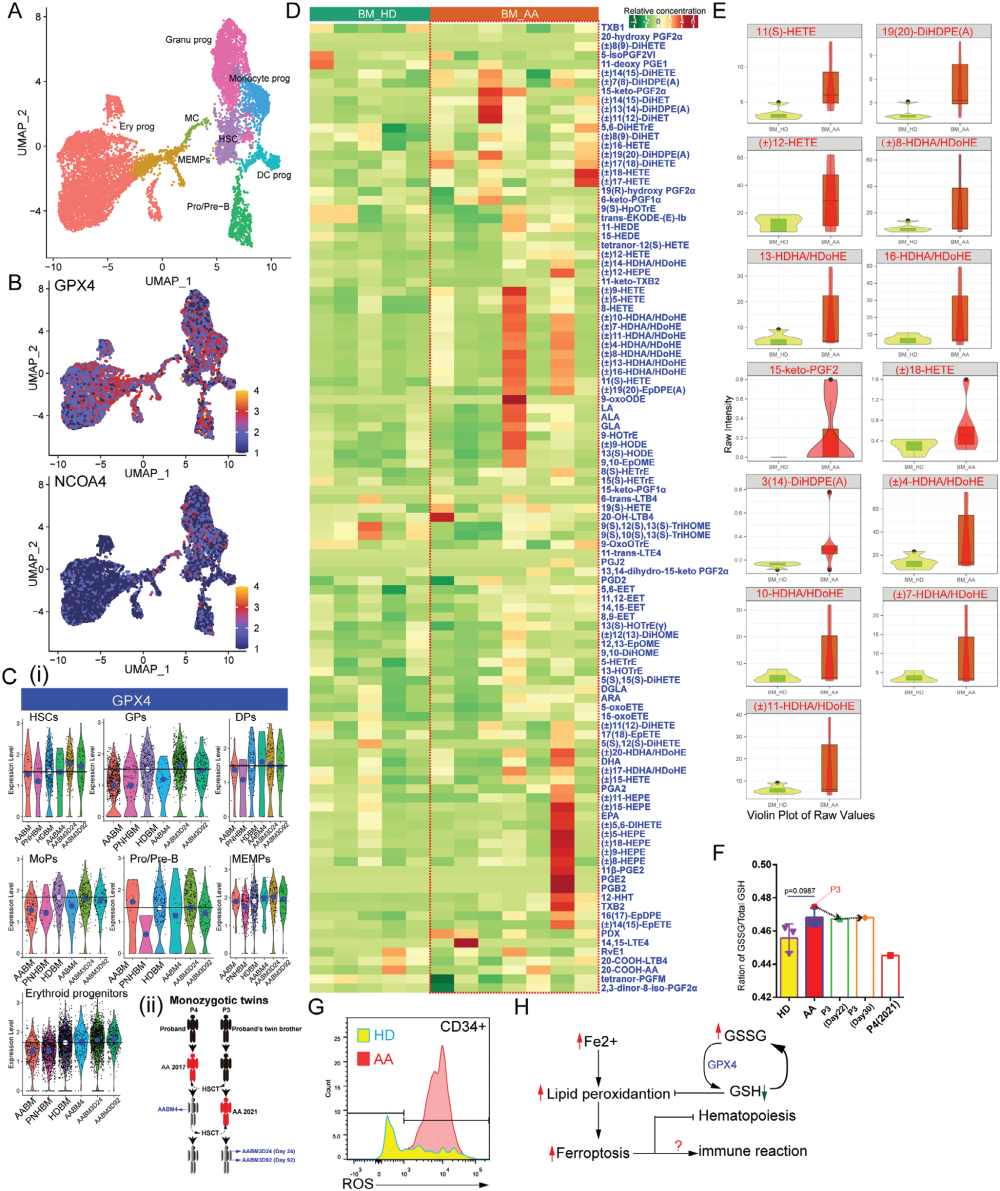
Ferroptosis impaired hematopoiesis by targeting HSPCs. A) UMAP plot of HSPCs and their lineage‐restricted progenitors from HDs and patients with AA. MEMPs, MK‐erythroid‐mast progenitors; MC, Mast cell. B) Feature Plots show the expression pattern of *GPX4* and *NCOA4* in HSPCs. C) Violin plots show the expression levels of *GPX4* per HSPC subpopulation in sample groups i). Large blue points indicate the average gene expression levels in each group. Large white points and black horizontal lines show the average gene expression levels in HDBM‐derived cells. The description of the monozygotic twins with AA ii). Before HSCT, residual BM samples (AABM4) were collected from the clinical examination of the cured proband (P4). After HSCT, BM samples were collected from the receptor (P3) at different time points (day 24, AABM3D24; day 92, AABM3D92). These samples were subjected to scRNA‐seq analysis. D) Heatmap showing the relative concentration of oxidized fatty acids per sample of BM plasma. E) Violin plots show the raw intensity of representative oxidized fatty acids. F) The GSSG/total GSH ratio to assess the oxidative stress of plasma. Black arrows showing the changes in the GSSG/total GSH ratio in P3‐derived samples during HSCT treatment. G) ROS levels of HSPCs from fresh samples as measured by Flow cytometry. H) Summary of increased ferroptosis sensitivity in HSPCs in patients with AA involving HSPC destruction.

Iron overload is a significant characteristic of patients with AA, and serum ferritin levels can be used as an index of iron levels.^[^ ^18^ ^]^ We measured the serum ferritin levels in patients with AA (Figure S6A, Supporting Information) and confirmed the upregulation of iron levels. We found that HSPCs were more sensitive to ferroptosis than stromal cells and mature hematopoietic cells (Figure S6B,C, Supporting Information). We extracted and reclustered HSPCs combined with lineage‐primed progenitor (**Figure** **6**A; Figure S6D, Supporting Information). Combined with changes in other ferroptosis‐related genes (Figure S6E,F, Supporting Information), the imbalance of *GPX4* and *NCOA4* among the various subpopulations suggested different sensitivities of ferroptosis in different subsets (Figure 6B). *GPX4* was highly expressed in HSPCs and progenitors (granulocyte progenitors (GPs), dendritic cell progenitors (DPs), monocyte progenitors (MoPs), and erythroid progenitors) of HD BM compared with that in AA samples (Figure 6C). After receiving HSPCs from identical twin brothers, *GPX4* expression was upregulated in various HSPCs and progenitor subsets.

The OFA levels are regarded as indicators of ferroptosis. OFA levels were increased in the BM plasma of patients with AA (Figure 6D). Several OFAs, including 11(S)‐HETE, 19(20)‐DiHDPE(A), (±)8‐HDHA/HDoHE, (±)12‐HETE, 13‐HDHA/HDoHE, 16‐HDHA/HDoHE, 15‐keto‐PGE2, (±)18‐HETE, 3(14)‐DiHDPE(A), (±)4‐HDHA/HDoHE, 10‐HDHA/HDoHE, (±)7‐HDHA/HDoHE, and (±)11‐HDHA/HDoHE, were increased in AA BM plasma (Figure 6E). As previously reported, ferroptosis leads to an increase in 11(S)‐HETE.^[^ ^19^ ^]^ Excess 15‐keto‐PGE2 induces ROS‐dependent cell death by reducing cellular glutathione levels and impairing the expression of solute carrier family seven member 11 (SLC7A11) and cystathionine gamma‐lyase (CTH).^[^ ^20^ ^]^ Principal component analysis (PCA) was applied to our OFA datasets, and a 3D scatter plot was used for visualization (Figure S6G, Supporting Information). The distribution of HD samples was highly concentrated, whereas that of AA samples was discrete. A higher GSSG/Total GSH ratio was observed in the AA group than those in the HD PB‐derived plasma samples (Figure 6F). AA CD34^+^ HSPCs showed higher ROS levels than that those in HD HSPCs; however, this phenomenon was not observed in CD3^+^ T‐cells, indicating that HSPCs lack antiferroptotic abilities (Figure 6G; Figure S6H, Supporting Information). Collectively, these results indicated that ferroptosis is a negative regulator of hematopoiesis in patients with AA (Figure 6H).

### 2.7 Tracing Dynamic Changes of Immune Rebalancing and Cell‐Fate in Monozygotic Twins with AA Undergoing Reciprocal Hematopoietic Stem Cell Transplantation (HSCT)

**Figure 7:**
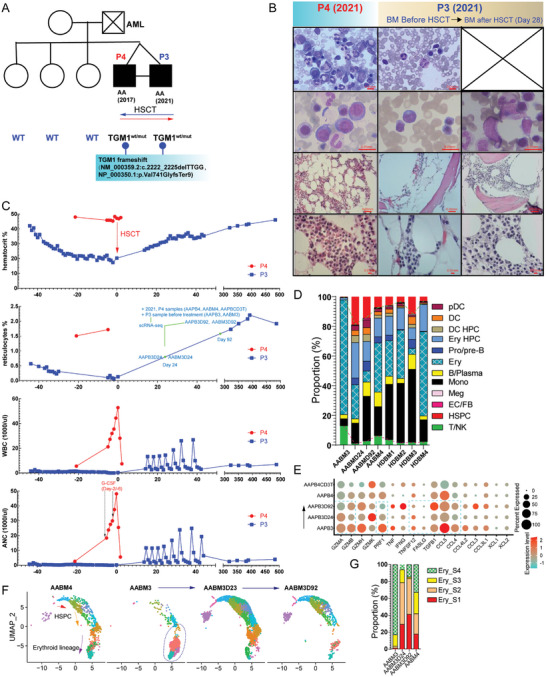
The description of a case of monozygotic twins with AA. A) Family pedigree. The shaded symbols denote the phenotype of AA (males, squares; females, circles). B) BM hematoxylin and eosin stain and BM aspirate smear of twins at different times. C) PB laboratory values, including hematocrit, percentage of reticulocytes, WBC count, and ANC, were plotted before and after HSCT. The red arrow on day 0 indicates the initiation of HSCT. D) The proportions of each subpopulation in each sample from the twins’ PBMC. E) The dot plot shows the expression of selected effectors in CD8^+^ T‐cells from the twins at different times. The dashed line rectangles indicate that effector genes were downregulated or upregulated during HSCT treatment. F) UMAP shows the distribution of erythroid differentiation stages in twins. G) The bar plot shows the proportions of the four erythroid stages in each of the twin's samples.

A 26‐year‐old proband (P4) of twins was diagnosed with AA and underwent HSCT for the HSPCs of the twin brother. After 4 years, the proband's twin brother (P3) was also diagnosed with AA. We examined the twins and their sisters for genetic mutations. We found a mutation site for *transglutaminase 1* (*TGM1*) in the twins, but not in their sisters (**Figure** **7**A). However, there was no evidence for an association between mutated *TGM1* and AA. The cured proband resolutely provided HSC to his brother. We investigated thriving BM hematopoiesis in his cured brother four weeks after HSCT (Figure 7B). The hematocrit, reticulocyte, white blood cell (WBC), and absolute neutrophil count (ANC) indices showed that the proband's HSC recovered the hematopoiesis of his brother (Figure 7C). This case report provides novel insights into the pathogenesis and treatment of AA. Fortunately, we obtained the twins' PBMC and BMMC scRNA‐seq datasets at different time points (Figure 7C). Restoration of hematopoiesis was accompanied by resting CD8^+^ T‐cells. After HSCT, the proportion of T/NK cells decreased and the proportion of HSPCs increased (Figure 7D). CD8^+^ T‐cells downregulated the expression of effector genes (Figure 7E), indicating that newly colonized HSCs temporarily interacted with CD8^+^ T‐cells. *TGFB1* is upregulated in CD8^+^ T‐cells. Dynamic changes in HSPCs and ferroptosis are shown in Figure 6. Erythropoiesis was restored by day 24 or earlier (Figure 7F). Early stages (S1/S2) of the erythroid lineage were supplemented to participate in erythropoiesis (Figure 7G). MC analysis showed that the frequencies of CD8^+^CD45RA^+^CCR7^−^ effector T, CD4^+^CD25^−^CXCR5^+^CCR4^−^ TFH, CD4^+^CD25^−^CXCR5^−^CXCR3^+^CCR6^+^ TH1‐like TH17, and CD4^+^CD25^−^CXCR5^−^CXCR3^−^CCR6^+^ TH17 cells decreased after HSCT (Figure S7, Supporting Information). The frequencies of pDC and FAS^−^CD34^+^ HSPCs increased. This case of monozygotic twins with AA provided a novel insight into the theories of “seed‐soil‐worms” in AA pathogenesis. Cases of twins with AA have been reported in other studies,^[^ ^21^, ^22^ ^]^ however, these analyses were descriptive. We provided high‐resolution scRNA‐seq datasets of twins at different time points during treatment to illustrate the etiology of AA based on HSPC destruction, abnormal BM microenvironments, hyperactivated immune responses, or a combination of these.

## 3 Discussion

Although AA is characterized by the destruction of HSPCs mediated by hyperactivated T‐cells,^[^ ^23^ ^]^ the heterogeneous pathology of AA presents challenges in eradicating pathogenic factors. Therefore, current treatment strategies may be more satisfactory.^[^ ^24^ ^]^ To delineate the cell atlas of BMMC and PBMC of patients with AA, we performed unbiased scRNA‐seq and aimed to investigate AA. The multiplex cytokine profile helped identify the extrinsic drivers of the immune responses. Notably, type 2 cytokines (IL‐4/6/10/13) combined with GM‐CSF and IL‐1β dominate the BM plasma samples, not IFN‐γ and TNF‐α. IL‐1β can enhance GM‐CSF production in TH1 and TH17, while GM‐CSF contributes to central nervous system inflammation.^[^ ^25^ ^]^ High IL‐4 and IL‐10 levels may contribute to the formation of autoantibody‐secreting plasma cells in patients with AA.^[^ ^26^, ^27^ ^]^ These results strongly suggest that activated CD4^+^ effectors (TH2 with TFH, and TH1‐like TH17) are crucial for the pathogenesis of AA, which is consistent with the results of previous studies.^[^ ^28^, ^29^ ^]^

The depletion of Vδ2 T‐cells and increased cytotoxicity are unique features in AA, consistent with acquired chronic pure red cell aplasia.^[^ ^30^ ^]^ FASLG expression is upregulated in CD8^+^ T‐cells during cell activation.^[^ ^31^ ^]^ FASLG inhibits normal hematopoiesis in myelodysplastic syndrome marrow.^[^ ^32^ ^]^ Our results support the notion that FAS‐FALG signaling is a therapeutic target for AA. Targeting TNFSF12 (TWEAK)‐TNFRSF12A(FN14) is another potential therapeutic strategy,^[^ ^33^ ^]^ that may help to rescue HSPCs and Tregs. The expression of *TNFSF10* mRNA was downregulated in almost all the cell types. Activated type I IFN signaling promotes TNFSF10 expression.^[^ ^34^ ^]^ Loss of IFN signaling may inhibit the expression of TNFSF10, which is consistent with our results regarding IFN‐response defects in patient‐derived cells. IFN‐response defects existed in all major lineages, including erythroid lineages, HSPCs, T/NK cells, myeloid cells, and B/plasma cells (Figure S8, Supporting Information), indicating that the IFN‐response is vital for immune homeostasis. Tconv cells expressed TNFRSF10D at a higher level in HDs than in patients with AA, indicating that normal T‐cells resist TRAIL‐mediated apoptosis. With the downregulation of TNFSF10 in patients with AA, TNFRSF10D was also downregulated, indicating that TRAIL‐triggered stress was defective, and autoreactive T‐cells could not be suppressed by TRAIL‐mediated apoptosis. Abnormal CXCR4 expression in Tconv cells,^[^ ^35^ ^]^ and the loss of CXCR4 expression in Tregs contribute to the infiltration of autoactivated T‐cells into the BM and impaired BM residence of Tregs. There is a strong correlation between the proportion of KIR^+^CD8^+^ T‐cells and disease severity in several autoimmune (such as multiple sclerosis, celiac disease, systemic lupus erythematosus, and rheumatoid arthritis) and infectious diseases (COVID‐19).^[^ ^36^ ^]^ Additionally, ex vivo expansion and reinfusion of KIR^+^CD8^+^ T‐cells may be an effective strategy for treating autoimmune diseases, including AA.

Iron overload is an adverse factor in patients with AA,^[^ ^37^ ^]^ which leads us to consider the relationship between dysfunctional iron homeostasis and ferroptosis. Our results identify ferroptosis as a critical driver of HSPCs destruction in patients with AA. *GSTT1* null genotype (absence of both alleles) is associated with a significantly increased risk of AA,^[^ ^38^ ^]^ providing evidence of reactive oxygen‐induced ferroptosis in patients with AA. *MYSM1* mutation, related to bone marrow failure syndrome 4 (OMIM #618 116), can drive ferroptosis in HSC.^[^ ^39^ ^]^ *MYSM1* was widely expressed in different HSPC subsets (Figure S9A, Supporting Information), and HD‐derived HSPC expressed higher levels of *MYSM1* than their AA counterparts (Figure S9B, Supporting Information), further confirming the role of ferroptosis in patients with AA. A previous report showed that excessive IL‐4/13 upregulated the expression of 12/15‐LOX,^[^ ^40^ ^]^ indicating that these cytokines may contribute to ferroptosis in HSPCs. Our study highlights that inhibiting ferroptosis using iron removal therapy may be useful for prolonging patient survival. Another mode of cell death, pyroptosis mediated by cytotoxic cells, is also highly likely to be involved in the disruption of hematopoiesis.

Herein, we report the case of monozygotic twins with AA, providing novel insights into the pathogenesis of AA. After HSCT with HSPCs in the recovered proband, the proband's twin brother underwent hematopoietic reconstruction. With the reconstruction of hematopoiesis, the ferroptosis sensitivity of HSPCs in the proband's twin brother was downregulated, suggesting that the recovery of iron homeostasis with the restart of erythropoiesis is important for HSPCs survival. The case of monozygotic twins with AA prompts insights into the theories of “seed‐soil‐worms” in AA pathogenesis.

Due to ethical limitations, we were unable to obtain sufficient BM samples from HDs, which may introduce potential bias to our findings. Further studies are necessary to increase the sample size. Although our study had many limitations, we provide a more comprehensive perspective on AA pathogenesis using unbiased scRNA‐seq and MC analyses. Trigger factors for AA remain poorly defined and can be controlled by regulating the immune network. In this study, we provided an unbiased atlas of hematopoiesis and immune regulation networks, to identify pathological immune subsets and possibilities for new strategies (Figure S10, Supporting Information). More fundamentally, our findings raise the question of whether manipulating context (collagen, laminin, arachidonic acid derivatives, ALCAM, GM‐CSF, IL1β, IL6, IL4, IL13, CCL2, PDGF‐BB, and FASLG), cell death (apoptosis, ferroptosis, and pyroptosis), innate immunity (monocytes, macrophages, DC, γδ T, and NK cells), or novel immune cell types (TH1‐like TH17 and KIR^+^CD8^+^ Treg) can be used as a strategy to weaken conventional T cell‐mediated hyperactivated immune response. We also reported many ignored factors, such as the TNFSF10, TNFSF12, and ZFP36 families, that offer opportunities for future clinical interventions.

## 4 Experimental Section

### Clinical Samples and scRNA‐Seq

Peripheral blood (PB) and BM were obtained from nine patients with AA, three patients with PNH, and four HDs (Table S1, Supporting Information), all recruited via the First Affiliated Hospital of Zhengzhou University (ZZU). Biospecimen collection protocols complied with the local guidelines and were approved by the Research and Clinical Trial Ethics Committee of the First Affiliated Hospital of ZZU (SS‐2018‐42, 2022‐KY‐0725, 2023‐KY‐0813, and 2023‐KY‐0830). The HD samples were obtained from donors of HSC collection who had no tumors, hematologic disorders, or other unfavorable factors after physical examination with routine blood examination, liver/kidney function tests, infectious disease screening, electrocardiographic examination, and color ultrasound examination. PB/BM samples from HDs and patients were collected in ethylenediaminetetraacetic acid (EDTA) anticoagulant tubes, and mononuclear cells (MNCs) were isolated using Ficoll‐Paque PLUS (Cat#17144003, Cytiva). A single‐cell suspension with 100 000 cells mL^−1^ concentration was prepared in PBS and loaded onto the microfluidic device. Libraries were constructed using the GEXSCOPER single‐Cell RNA‐Library Kit (Cat#5180012, Singleron). Individual libraries were sequenced on an Illumina HiSeq X. system, *CeleScope* (https://github.com/singleron‐RD/CeleScope) *v1.9.0 pipeline*, and *Cutadapt* (v1.17) was used to obtain gene expression matrices from raw reads. We then used *STAR* (v2.6.1a) to map the reads to the reference genome *GRCh38* (ensemble version 92 annotation). The expression matrix files were generated by *feature‐Counts* (v2.0.1)

### Analysis of scRNA‐Seq Datasets

Seurat package was used for dimension reduction, cell clustering, and differential gene expression analyses. *CellChat* was used for cell communication.^[^ ^41^ ^]^ *Clusterprofiler* package was used to perform GO analysis of DEGs between different clusters.^[^ ^42^ ^]^ *Fgsea* package was used to perform GSE analysis of Treg clusters.^[^ ^43^ ^]^ These data sets can be downloaded from figshare (https://figshare.com/s/b1d863820afa167fe040).

### scRNA‐Seq Analysis of all Cells from Patients with AA/PNH and Healthy Donors

“*CreateSeuratObject*” function with the following criteria: one gene was expressed in at least three cells, and at least 200 genes were detected in one cell. The cells were then filtered with a gene‐expression number per cell between 200 and 10 000 and a mitochondrial percentage < 25. Further analyses, including normalization, scaling, cell clustering, and identification of marker genes, were performed using *Seurat* (version 4)^[^ ^44^ ^]^ (Table S3, Supporting Information). The “*RunHarmony*” function was used to remove batch effect based on the group.by.vars = “*orig.ident*”. Clusters were calculated using the *FindClusters* function with a resolution of 1.5 and visualized using the UMAP dimensional reduction method. The lineage features of the primary clusters were identified by marker genes (Mono: *CD14* and *FCGR3A*; T/NK: *CD3D*, *CD8A*, and *IL7R*; B/Plasma: *MS4A1*, *CD19*, *XBP1*, and *MZB1*; Ery: *GATA1*, *HBD*, *HBA1*, and *HBA2*; Pro/pre‐B: *IL7R*, *CD34*, *MS4A1*, and *CD19*; Granulocytes: *CEACAM8*, *CD177*, *S100A8*, and *S100A10*; Erythroid progenitors: *CD34*, *HOXA5*, *HOXA9*, *HOXA10*, *GATA1*, *HBD*, *HBA1*, and *HBA2*; HSPCs: *CD34*,*FLT3 HOXA5*, *HOXA9*, and *HOXA10*; DC progenitors: *CD34*, *HOXA5*, *HOXA9*, *HOXA10*, *FLT3*, *CD1C*, *CLEC10A*, *HLA‐DQA1*, *HLA‐DPB1*, and *HLA‐DRA*; DC: *CD1C*, *CLEC10A*, *HLA‐DQA1*, *HLA‐DPB1*, and *HLA‐DRA*; pDC: *JCHAIN*, *IL3RA*, *IRF8*, and *IRF7*; Megakaryocyte: *PPBP, PF4*, and *GATA2*; EC/FB: *APOE*, *VCAM1*, *DCN*, *COL1A2*, and *COL14A1*) as presented in Figure S1A (Supporting Information), and 45 small clusters were merged into 13 clusters based on lineage features (Figure 1B; Figure S1A, Supporting Information). Representative samples were visualized using split.by = “orig.ident”. A *CellChat* object was generated using createCellChat(object = *SeuratObject@assays$RNA@data*, meta = *SeuratObject@meta.data*, group.by = “*CellType*”). We used *CellChatDB.human* as the basis for the ligand‐receptor analysis. We followed the *CellChat* protocol to visualize cell communication.^[^ ^41^ ^]^ The *AddModuleScore* command from Seurat was used to calculate the average expression for each molecular subgroup (ferroptosis suppressor gene set [Table S4, Supporting Information] and ferroptosis driver gene set [Table S4])^[^ ^45^, ^46^ ^]^ and the results were visualized in ViolinPlot and FeaturePlot.

### scRNA‐Seq Analysis of CD8^+^ T‐cells

We extracted CD8^+^ T‐cells by performing subset(Object, CD8A>0 | CD8B>0,slot = “counts”), and then reclustered these cells. Next, we removed clusters without features of CD8^+^ T‐cells, which expressing CD8A/CD8B but significantly presenting other lineage‐specific markers (*CD4*, *FOXP3*, *TRGC2, TRGC1*, *NCAM1*, *CD14*, *CEACAM8*, *CD177*, *MS4A1*, *CD19*, *GATA1*, *HBD*, *HBA1*, *HBA2*, *CD1C*, *JCHAIN*, *PPBP*, *PF4*, *APOE*, *VCAM1*, *DCN*, *COL1A2*, and *COL14A1*). We got 18 primary clusters by performing *FindClusters*(*resolution* = *1.5*). The subpopulation features of the primary clusters were identified using marker genes (Proliferating CD8^+^ T: *TYMS*, *MKI67*, and *TOP2A*; MAIT: *TRAV1‐2*, *KLRB1*, *SLC4A10*, *CEBPD*, *PDCD4*, *IFNGR1*, *RORA*, *CXCR4*, *TNFRSF25*, *DPP4*, and *CXCR6*; Effector CD8^+^ T: *GZMB*, *GZMH*, *CX3CR1*, *FCGR3A*, *FGFBP2*, *FCRL6*, *PRSS23*, and *FGR*; Memory CD8^+^ T: *IL7R*, *LEF1*, *PABPC1*, *LTB*, *TCF7*, *SELL*, *NELL2*, *GPR171*, and *FTH1*; Effector‐memory CD8^+^ T: *GZMB*, *GZMH*, *CX3CR1*, *FCGR3A*, *FGFBP2*, *FCRL6*, *PRSS23*, *FGR*, *IL7R*, *LEF1*, *PABPC1*, *LTB*, *TCF7*, *SELL*, *NELL2*, *GPR171*, and *FTH1*; Naïve CD8^+^ T: *LRRN3*, *MYC*, *CCR7*, *ACTN1*, *PASK*, *CD55*, and *PIM2*; activated IFN CD8^+^ T: *IFIT1*, *RSAD2*, *IFIT3*, *MX1*, *OAS1*, *ISG15*, *IFI44L*, *IFIT2*, *GZMB*, *GZMH*, *CX3CR1*, *FCGR3A*, *FGFBP2*, *FCRL6*, *PRSS23*, and *FGR*; Resting IFN CD8^+^ T: *IFIT1*, *RSAD2*, *IFIT3*, *MX1*, *OAS1*, *ISG15*, *IFI44L*, *IFIT2*, *LRRN3*, *MYC*, *CCR7*, *ACTN1*, *PASK*, *CD55*, and *PIM2*; KIR^+^CD8^+^ T: *KIR3DL1*, *KIR3DX1*, *KIR2DL1*, *KIR2DL4*, *KIR2DS4*, *KIR3DL2*, and *KIR2DL3*). Small clusters were then merged into nine clusters based on lineage features (Figure 2A; Figure S2A, Supporting Information). We used the FindMarkers function to identify the differentially expressed genes. The cluster Profiler package was used to perform GO analysis of differential genes between the KIR^+^CD8^+^ T subset and effector CD8^+^ T subset.

### scRNA‐Seq Analysis of CD4^+^ T‐cells

We excluded *CD8A*/*CD8B*/*TRDC*‐expressing cells and reclustered the remaining cells. Next, we removed clusters without features of CD4^+^ T‐cells. The subpopulation features of the primary clusters were identified using marker genes (resting subset: *SELL*, *LEF1*, *CD7*, *CCR7*, *PECAM1*, and *TCF7*; Treg: *FXOP3*, *IL2RA*, *IKZF2*, and *CTLA4*; proliferating subset: *TYMS* and *MKI67*; effector subset: *RORC*, *CCR6*, *IL23R*, *GATA3*, *CCR4*, *IL4R*, *PTGDR2*, *CXCR3*, *EOMES*, *RUNX3*, *PDCD1*, *TBX21*, *IFNG*, *ADGRG1*, *CX3CR1*, *GZMB*, *PRF1*, *GNLY*, *GZMK*, *TNF*, *BHLHE40*, *CCL5*, and *GZMA*; IFN subset: *IFIT3*, *IFI44L*, *ISG15*, and *MX1*). Small clusters were merged into five based on lineage features (Figure S3A,B, Supporting Information). Scores of TH subpopulation features (Table S5, Supporting Information) were calculated using the Seurat *AddModuleScore* function. We extracted CD4^+^ T‐cells from AABM, AAPB, HDBM, and HDPB and then merged them with those sorted from a scRNA‐seq dataset of healthy donor‐derived bone marrow CD3^+^CD4^+^CD8^−^CD127^−/low^CD25^+^ Treg cells. The subpopulation features of the primary clusters were identified using marker genes (resting subset: *SELL*, *LEF1*, *CD7*, *CCR7*, and *TCF7*; Activated Treg: *HLA‐DQB1*, *HLA‐DRB1*, *HLA‐DQA1, HLA‐DRB5*, *HLA‐DMA, HLA‐DRA*, *HLA‐DRB6*, *HLA‐C*, *HLA‐F*, *HLA‐A*, and *HLA‐B*; MKI67+ Treg: *MKI67* and *TYMS*). Small clusters were merged into four based on lineage features (Figure S3E, Supporting Information). We used the *fgsea* package to assess the score of the interested pathway or gene sets.

### scRNA‐Seq Analysis of NK/NKT/γδ T‐cells

We extracted T/NK cells (Figure 1B) and removed the clusters of conventional CD4^+^/CD8^+^ T‐cells and Treg cells. Clusters were calculated using the *FindClusters* function with a resolution of 2.5 and visualized using the UMAP dimensional reduction method. Lineage features of the primary clusters were identified using marker genes (*TRGC2*, *TRGC1*, *KLRF1*, *CD3E*, *CD247*, *NCAM1*, *KLRB1*, *FCGR3A*, and *KLRC2*). Small clusters were merged into three clusters based on their lineage features. The scores for interested δ1 and δ2 T features (Table S6, Supporting Information) were calculated using the Seurat *AddModuleScore* function.

### scRNA‐Seq Analysis of B Cells

We excluded *CD3D*/*CD3E*/*CD3G*/*TRAC*/*CD8A*/*CD8B*/*TRDC*‐expressing cells and reclustered the remaining cells. Next, we removed the other clusters without features of B and plasma cells. The lineage features of the primary clusters were identified using marker genes (naïve B cells: *FCER2*, *TCL1A*, and *IL4R*; intermediate B cells: *FCER2*, *TCL1A, IL4R, CD27, AIM2*, and *TNFRSF13B;* memory B cells: *CD27, AIM2*, and *TNFRSF13B*; Proliferating plasma cells: *MKI67, TOP2A, MZB1, SDC1*, and *CD38*; IGHA^+^ plasma cells: *MZB1, SDC1, CD38, IGHA1*, and *IGHA2*; IGHG^+^ plasma cells: *MZB1, SDC1, CD38, IGHG1, IGHG2, IGHG3*, and *IGHG4*). Small clusters were merged into three clusters based on their lineage features.

### scRNA‐Seq Analysis of Erythroid Lineage

HSPCs and erythroid lineages were extracted from all cells. We then removed other potential lineages. Representative samples were visualized using *split.by = “orig.ident”*. Next, we removed the HSC population and reclustered erythroid lineages. These erythroid lineages are divided into four stages. We extracted erythroid and NK/T lineages, and reclustered these cells. The cells were then divided into 12 clusters (Ery_S1/S2/S3/S4, γδ T, MAIT, NK, NKT, CD4^+^ Treg, CD4^+^ Tconv, CD8^+^ T, and proliferating T). We used the CellChat package to visualize the communication between erythroid lineages and immune cell types.

### scRNA‐Seq Analysis of pDC and Myeloid Cells:

We extracted pDC and myeloid cells from all cells. We then removed other potential lineages. The lineage features of the primary clusters were identified using marker genes (neutrophil: *CXCR2*, *CXCR4*, *CEACAM8*, *CD177*, and *S100A12*; monocytes: *CD14* and *FCGR3A*; macrophages: *C1QC*, *C1QB*, and *C1QA*; DC: *CLEC9A*, *ITGAE*, *ITGB7*, *CD74*, *FCER1A*, *HLA‐DRA*, *HLA‐DPB1*, *CD1C*, *HLA‐DQA1*, and *HLA‐DQB1*; pDC: *LRRC26*, *MYBL2*, *SHD*, *COL26A1*, *PACSIN1*, *KIRREL3*, *KRT5*, *KCNA5*, and *NLRP7*). Clusters were extracted using the subset function. The DEGs of the different clusters were obtained using the *FindMarkers* function. Based on these DEGs, the clusterProfiler package was used for the GO analysis. We used the *CellChat* package to visualize cell communication between the pDC/myeloid and NK/T lineages.

### scRNA‐Seq Analysis of HSPCs

We extracted CD34^high^ and CD34^low^ populations, but not CD34^−^ populations. We then reclustered the remaining cells and removed other clusters without progenitor features. The lineage features of the primary clusters were identified using marker genes, which are provided in Figure S6D (Supporting Information).

### Mass Spectrometry of OFAs Metabolic Profiles

Plasma samples were isolated from BM by centrifugation at 400 × g for 10 min. Platelets were removed from BM plasma by centrifugation at 2000 × g for 15 min. These BM plasma samples were stored at −80 °C. These samples were thawed on ice, and 200 µl pure‐Methanol solution containing internal standard was added into 100 µl BM plasma. The mixture was vortexed for 5 min and precipitated at a low temperature (−20 °C) for 30 min to remove protein. And then, samples were centrifuged at 12 000 rpm for 10 min (4 °C), and all supernatant was collected and transferred. Repeat the extraction once and combine the supernatants. The eicosanoids in supernatants were extracted using Poly‐Sery MAX SPE columns (ANPEL). Before analysis, the eluent was dried under a vacuum and redissolved in 100 µl of methanol/water (1:1,v/v) for UPLC/MS/MS analysis. The sample extracts were analyzed using an LC‐ESI‐MS/MS system (UPLC, ExionLC AD; MS, QTRAP 6500+ System). The analytical conditions were as follows, HPLC: column, Waters ACQUITY UPLC HSS T3 C18 (100 mm × 2.1 mm i.d. 1.8 µm); solvent system, water with 0.04% acetic acid (A), acetonitrile with 0.04% acetic acid (B); The gradient was 0–2.0 min from 0.1% to 30%B; 2.0–4.0 min to 50% B; 4.0–5.5 min to 99% B, which was maintained for 1.5 min; and 6.0–7.0 min reduced to 0.1% B and maintained for 3.0 min. flow rate, 0.4 mL mi^−1^n; temperature, 40 °C; injection volume: 10 µl. Linear ion trap (LIT) and triple quadrupole (QQQ) scans were acquired on a triple quadrupole‐linear ion trap mass spectrometer (QTRAP), QTRAP 6500+ LC‐MS/MS System, equipped with an ESI Turbo Ion‐Spray interface, operating in negative ion mode and controlled by Analyst 1.6.3 software (Sciex). The ESI source operation parameters were: an ion source, ESI‐; source temperature 550 °C; ion spray voltage (IS) −4500 V; curtain gas (CUR) was set at 35 psi, respectively. Eicosanoids were analyzed using scheduled multiple reaction monitoring (MRM). Data acquisitions were performed using Analyst 1.6.3 software (Sciex). Multiquant 3.0.3 software (Sciex) was used to quantify all metabolites. Mass spectrometer parameters were used with further DP and CE optimization, including the declustering potentials (DP) and collision energies (CE) for individual MRM transitions. A specific set of MRM transitions were monitored for each period according to the metabolites eluted within this period. Data were analyzed using *R* software.

### Mass Spectrometric Analysis

Cells were washed with LunaStain cell staining buffer (Polaris Biology) and stained with 10 µl of Cisplatin reagent (Polaris Biology) at room temperature for 5 min. The cells were washed with LunaStain cell staining buffer (Polaris Biology) and stained with 5 µl of Fc block (Biolegend) for 10 min and the heavy metal‐labeled membrane antibody mixtures for 30 min at room temperature (Table S7, Supporting Information). Cells were washed twice with LunaStain cell staining buffer (Polaris Biology) and stained with Ir‐DNA intercalator reagent (Polaris Biology) for 10 min. After staining, cells were washed and adjusted to 1 million cells per milliliter in LunaAcq cell acquisition solution (Polaris Biology) with 20 µl of SureBits element calibration beads (Polaris Biology). Cell acquisition was performed using a mass cytometer at 300 events per second (StarionX1, Polaris Biology). After the acquisition, the mass cytometry data were normalized and converted into standard FSC 3.0 files (StarionX1, Polaris Biology). Manual gating was performed using FlowJo software (BD Biosciences). These data sets can be downloaded from figshare (https://figshare.com/s/3a164998e3d909adfd4e).

### High‐Sensitivity Multiplex Cytokine Assays

BM plasma samples with platelet removal were stored at −80 °C and thawed on ice. Some patients with AA (P13, P6, P15, P17, P20, P21, P22, P23, P24, P25, P27, P28, P29, P3, P30, P33, P34, P35, P36, P37, P38, P5, P42, P43, P44, P46, P9, P47, P48, P49, P50, P51, P52, P53, P54, and P55) and one healthy donor (HSCT donor, HD6) were treated with G‐CSF before BM collection (Table S8, Supporting Information). Multiplex assays were performed using the bead‐based immunoassay approach Bio‐Plex Pro Human Cytokine 27‐plex Assay (Bio‐Rad) on a Bio‐Plex 200 System (Bio‐Rad). The data were analyzed using *R* software, and the heatmap was calculated and visualized using *log2* (raw data+1).

### GSSG Detection and ROS Detection

BM plasma samples with platelet removal were stored at −80 °C and thawed on ice. The GSSG and total GSH concentrations were measured using the GSH/GSSG Ratio Detection Assay Kit (Beyotime). The level of ROS in BM plasma with platelet removal was detected using a Reactive Oxygen Species Assay Kit (Beyotime) according to the manufacturer's instructions. Fresh PB and BM samples (< 1 h) were treated with ACK buffer to remove red blood cells and re‐suspended at 1 × 10^7^ cells. The positive control cells were treated with Rosup (1:2000) for 20 min. Positive control and target samples were treated by DCFH‐DA (1:2000) at 37 °C for 30 min. The cells were washed, and Zombie NIR (BioLegend) was added to label the dead cells. 0.5 µl Biotin anti‐human CD34 antibody (BioLegend) was added to the washed cells and incubated for 30 min. 0.5 µl APC‐Streptavidin (BioLegend) and 1 µL PE anti‐human CD3 Antibody (BioLegend) were added to the washed cells and incubated for 15 min. The samples were washed, and analyzed using an Aria II.

### Flow Cytometry (FCM) Analysis

PB and BM samples from HDs and AA patients were collected in EDTA anticoagulant tubes and stored at 4 °C. PBMCs and BMMCs were isolated by density gradient centrifugation using Ficoll‐Paque PLUS within 24 h. PBMCs and BMMCs were blocked using FcR (CD16/32) Blocking Reagent (Miltenyi), and stained with the following antibodies/reagents: PE anti‐human CD158f (KIR2DL5) antibody (Biolegend), PE anti‐human CD158e1 (KIR3DL1, NKB1) antibody, PE mouse anti‐Human CD158a (BD Pharmingen), PE mouse anti‐human CD158b (BD Pharmingen), APC anti‐human TCRγ/δ (Biolegend), Biotin anti‐humanTCRvδ2 (Biolegend), APC/Cyanine7 streptavidin (Biolegend), human KIR3DL2/CD158k PE‐conjugated antibody (R&D), anti‐humanCD261(DR4)‐APC (Miltenyi), FITC anti‐human CD8a antibody (Biolegend), APC‐Cy7 anti‐human CD4 antibody (Biolegend), Percp‐Cy5.5 anti‐human CD25 antibody (Biolegend), PE‐Cy7 anti‐human CD127 antibody (eBioscience), PE/Cyanine7 Streptavidin (Biolegend), PerCP/Cyanine5.5 anti‐human CD19 (BioLegend), PE‐Cy7 anti‐human CD3 antibody (BioLegend), APC anti‐human TCRγ/δ antibody (BioLegend), and PE anti‐human CD184 (CXCR4) antibody (BioLegend). The cells were resuspended in 400 µl 0.1 µg mL^−1^ DAPI solution (Salarbio). Treg cells (CD8a^−^CD4^+^CD127^−/low^CD25^+^) were sorted using an Arial II (BD Biosciences).

### Detection of Apoptotic and Dead Cells

PBMC samples were isolated from fresh peripheral blood samples of patients with AA and healthy donors within 2 h of collection (AA, n = 5; HD, n = 4). PBMCs were blocked with FcR (CD16/32) Blocking Reagent, and stained with PE anti‐human CD19 antibody (BD). The fluorogenic peptide Apotracker Green (BioLegend) and DAPI were used to detect apoptotic and dead cells. These samples were analyzed using an Aria II. Apoptotic and dead cells were defined as DAPI^+^ and Apotracker Green^+^ cells.

Fresh PB samples from three HDs were collected in EDTA anticoagulant tubes, and PBMCs were isolated using Ficoll‐Paque PLUS. A single‐cell suspension with 1 000 000 cells mL^−1^ concentration was prepared in RPMI 10% human autogenous plasma. FASLG protein (100 ng mL^−1^) (novoprotein) was supplemented into the cell suspension at different time points (0, 12, and 22 h). After 24 hours, cells were stained with Treg‐related antibodies, Zombie NIR Fixable Viability Kit (Biolegend), and fluorogenic peptide Apotracker Green, and assessed for apoptosis by flow cytometry.

## Conflict of Interest

The authors declare no conflict of interest.

## Author Contributions

R.Q.G., Y.M.L., Z.X.J., and Y.P.S. performed conceptualization. R.Q.G., L.N.S., P.T., L.L., R.G., S.Y.W., K.T.Y.J.J.K., M.C.Q., and Z.L.B performed methodology. R.Q.G. and Y.M.L. performed investigation. R.Q.G. and Y.M.L. performed writing—original draft. R.Q.G. and Y.M.L. performed writing. All authors Reviewed and edited the manuscript. R.Q.G., Z.L.B., and S.Y.W. performed Funding acquisition. R.Q.G., L.N.S., P.T., and Y.M.L. performed Resources. R.Q.G., Y.P.S., Z.X.J., and Y.M.L. performed Supervision.

## Supporting information

Supporting Information

Supplemental Table 1

Supplemental Table 2

Supplemental Table 3

Supplemental Table 4

Supplemental Table 5

Supplemental Table 6

Supplemental Table 7

Supplemental Table 8

## Data Availability

The data that support the findings of this study are available from the corresponding author upon reasonable request.

## References

[1] J. Huuhtanen , S. Lundgren , M. A. Keränen , X. Feng , C. M. Kerr , E. Jokinen , M. Heinonen , P. Savola , T. Kelkka , F. Ebeling , G. L. Ryland , L. Fox , P. Blombery , E. H. Lindberg , N. S. Young , J. P. Maciejewski , H. Lähdesmäki , S. Mustjoki , Blood. 2019, 134, 108.31296544

[2] C. Zhu , Y. Lian , C. Wang , P. Wu , X. Li , Y. Gao , S. Fan , L. Ai , L. Fang , H. Pan , T. Cheng , J. Shi , P. Zhu , Blood. 2021, 138, 23. 33763704 10.1182/blood.2020008966 PMC8349468

[3] H. Tonglin , Z. Yanna , Y. Xiaoling , G. Ruilan , Y. Liming , Front Genet. 2021, 12, 745483. 35046994 10.3389/fgene.2021.745483 PMC8762313

[4] J. N. P. Smith , V. S. Kanwar , K. C. Macnamara , Front. Immunol. 2016, 7, 330. 27621733 10.3389/fimmu.2016.00330 PMC5002897

[5] J. Kuruvilla , H. A. Leitch , L. M. Vickars , P. F. Galbraith , C. H. Li , S. Al‐Saab , S. C. Naiman , Eur J Haematol. 2003, 71, 396. 14667206 10.1034/j.1600-0609.2003.00115.x

[6] M. Medinger , B. Drexler , C. Lengerke , J. Passweg , Front. Oncol. 2018, 8, 587. 30568919 10.3389/fonc.2018.00587 PMC6290278

[7] Y. Zaimoku , B. A. Patel , S. Kajigaya , X. Feng , L. Alemu , D. Quinones Raffo , E. M. Groarke , N. S. Young , Br. J. Haematol. 2020, 190, 610. 32311088 10.1111/bjh.16651 PMC7496711

[8] C. Liu , Y. Chen , D. Lu , B. Liu , T. Zhang , L. Deng , Z. Liu , C. Zhong , R. Fu , Clin. Transl. Med. 2022, 12, e1092. 36471484 10.1002/ctm2.1092 PMC9722964

[9] M. Liu , T. Liu , W. T. Meng , H. L. Zhu , X. Cui , Zhongguo Shi Yan Xue Ye Xue Za Zhi 2007, 15, 142. 17490541

[10] W. Sun , Z. Wu , Z. Lin , M. Hollinger , J. Chen , X. Feng , N. S. Young , Blood. 2018, 132, 2730. 30361263 10.1182/blood-2018-05-844928 PMC6307988

[11] P. V. Kharchenko , Nat. Methods. 2021, 18, 723. 34155396 10.1038/s41592-021-01171-x

[12] J. Li , M. Zaslavsky , Y. Su , J. Guo , M. J. Sikora , V. Van Unen , A. Christophersen , S.‐H. Chiou , L. Chen , J. Li , X. Ji , J. Wilhelmy , A. M. Mcsween , B. A. Palanski , V. V. A. Mallajosyula , N. A. Bracey , G. K. R. Dhondalay , K. Bhamidipati , J. Pai , L. B. Kipp , J. E. Dunn , S. L. Hauser , J. R. Oksenberg , A. T. Satpathy , W. H. Robinson , C. L. Dekker , L. M. Steinmetz , C. Khosla , P. J. Utz , L. M. Sollid , et al., Science. 2022, 376, eabi9591. 35258337 10.1126/science.abi9591 PMC8995031

[13] Z. Zhang , Y. Zhang , S. Xia , Q. Kong , S. Li , X. Liu , C. Junqueira , K. F. Meza‐Sosa , T. M. Y. Mok , J. Ansara , S. Sengupta , Y. Yao , H. Wu , J. Lieberman , Nature. 2020, 579, 415. 32188940 10.1038/s41586-020-2071-9 PMC7123794

[14] U. Fischer , C. Ruckert , B. Hubner , O. Eckermann , V. Binder , T. Bakchoul , F. R. Schuster , S. Merk , H.-U. Klein , M. Führer , M. Dugas , A. Borkhardt , Haematologica. 2012, 97, 1304. 22315490 10.3324/haematol.2011.056705 PMC3436230

[15] G. Pizzolato , H. Kaminski , M. Tosolini , D.‐M. Franchini , F. Pont , F. Martins , C. Valle , D. Labourdette , S. Cadot , A. Quillet‐Mary , M. Poupot , C. Laurent , L. Ysebaert , S. Meraviglia , F. Dieli , P. Merville , P. Milpied , J. Déchanet‐Merville , J.‐J. Fournié , Proc. Natl. Acad. Sci. U. S. A. 2019, 116, 11906. 31118283 10.1073/pnas.1818488116 PMC6576116

[16] S. Sivori , P. Vacca , G. Del Zotto , E. Munari , M. C. Mingari , L. Moretta , Cell Mol. Immunol. 2019, 16, 430. 30778167 10.1038/s41423-019-0206-4 PMC6474200

[17] M. Krockenberger , Y. Dombrowski , C. Weidler , M. Ossadnik , A. Honig , S. Hausler , H. Voigt , J. R. C. Becker , L. Leng , A. Steinle , M. Weller , R. Bucala , J. Dietl , J. R. Wischhusen , J. Immunol. 2008, 180, 7338. 18490733 10.4049/jimmunol.180.11.7338 PMC3607742

[18] W. Yang , X. Zhao , G. He , H. Chang , B. Han , S. Gao , S. Wang , T. Chen , F. Li , Y. Wang , X. Ge , R. Fu , Z. Ge , Y. Li , H. Liu , X. Liu , M. Miao , L. Zhang , F. Zhang , Ann. Hematol. 2022, 101, 2611. 36220881 10.1007/s00277-022-04968-8

[19] J. P. Friedmann Angeli , M. Schneider , B. Proneth , Y. Y. Tyurina , V. A. Tyurin , V. J. Hammond , N. Herbach , M. Aichler , A. Walch , E. Eggenhofer , D. Basavarajappa , O. Rådmark , S. Kobayashi , T. Seibt , H. Beck , F. Neff , I. Esposito , R. Wanke , H. Förster , O. Yefremova , M. Heinrichmeyer , G. W. Bornkamm , E. K. Geissler , S. B. Thomas , B. R. Stockwell , V. B. O'Donnell , V. E. Kagan , J. A. Schick , M. Conrad , Nat. Cell Biol. 2014, 16, 1180. 25402683 10.1038/ncb3064 PMC4894846

[20] E. Y. Chang , Y.‐C. Chang , C.‐T. Shun , Y.‐W. Tien , S.‐H. Tsai , S.‐W. Hee , I.‐J. Chen , L.‐M. Chuang , PLoS One. 2016, 11, e0147390. 26820738 10.1371/journal.pone.0147390 PMC4731085

[21] P. T. Vo , J. Pantin , C. Ramos , L. Cook , E. Cho , R. Kurlander , H. Khuu , J. Barrett , S. Leitman , R. W. Childs , J. Hematol. Oncol. 2015, 8, 78. 26113077 10.1186/s13045-015-0173-x PMC4487559

[22] R. Gale , W. Hinterberger , N. S. Young , A. Gennery , C. Dvorak , K. Hebert , M. Heim , L. Broglie , M. Eapen , Res Sq. 2023. 10.1038/s41375-023-01892-2 PMC10353698 37106162

[23] J. M. Rosenberg , J. M. Peters , T. Hughes , C. A. Lareau , L. S. Ludwig , L. R. Massoth , C. Austin‐Tse , H. L. Rehm , B. Bryson , Y.‐B. Chen , A. Regev , A. K. Shalek , S. M. Fortune , D. B. Sykes , Med. 2022, 3, 42. 35590143 10.1016/j.medj.2021.12.003 PMC9123284

[24] D. V. Babushok , Blood. 2022, 139, 1. 34989776 10.1182/blood.2021014046

[25] M. El‐Behi , B. Ciric , H. Dai , Y. Yan , M. Cullimore , F. Safavi , G.‐X. Zhang , B. N. Dittel , A. Rostami , Nat. Immunol. 2011, 12, 568. 21516111 10.1038/ni.2031 PMC3116521

[26] T. Kelkka , M. Tyster , S. Lundgren , X. Feng , C. Kerr , K. Hosokawa , J. Huuhtanen , M. Keränen , B. Patel , T. Kawakami , Y. Maeda , O. Nieminen , T. Kasanen , P. Aronen , B. Yadav , H. Rajala , H. Nakazawa , T. Jaatinen , E. Hellström‐Lindberg , S. Ogawa , F. Ishida , H. Nishikawa , S. Nakao , J. Maciejewski , N. S. Young , S. Mustjoki , Leukemia. 2022, 36, 2317. 35927326 10.1038/s41375-022-01654-6 PMC9417997

[27] F. Franke , G. A. Kirchenbaum , S. Kuerten , P. V. Lehmann , Cells. 2020, 9, 433. 32069813 10.3390/cells9020433 PMC7072853

[28] J. Zhang , T. Liu , Y. Chang , Y. Duan , C. Liu , X. Chen , C. Tao , X. Zhu , Y. Zhang , Blood. 2022, 140, 475.

[29] S. Kordasti , J. Marsh , S. Al‐Khan , J. Jiang , A. Smith , A. Mohamedali , P. P. Abellan , C. Veen , B. Costantini , A. G. Kulasekararaj , N. Benson‐Quarm , T. Seidl , S. A. Mian , F. Farzaneh , G. J. Mufti , Blood. 2012, 119, 2033. 22138514 10.1182/blood-2011-08-368308

[30] M. Yoshihiro , M. Hirokawa , N. Fujishima , Y. Abe , M. Fujishima , Y.‐M. Guo , K. Ubukawa , L. Jiajia , T. Yoshioka , Y. Kameoka , H. Saitoh , H. Tagawa , N. Takahashi , K. Sawada , Blood. 2011, 118, 3429.

[31] G. Flores‐Mendoza , N. Rodríguez‐Rodríguez , R. M. Rubio , I. K. Madera‐Salcedo , F. Rosetti , J. C. Crispín , Front. immunol. 2021, 12, 635862. 33841416 10.3389/fimmu.2021.635862 PMC8024570

[32] H. J. Deeg , C. Beckham , M. R. Loken , E. Bryant , M. Lesnikova , H. M. Shulman , T. Gooley , Leuk Lymphoma. 2000, 37, 405. 10752992 10.3109/10428190009089441

[33] M. Chopra , A. Brandl , D. Siegmund , A. Mottok , V. Schäfer , M. Biehl , S. Kraus , C. A. Bäuerlein , M. Ritz , K. Mattenheimer , S. Schwinn , A. Seher , T. Grabinger , H. Einsele , A. Rosenwald , T. Brunner , A. Beilhack , H. Wajant , Blood. 2015, 126, 437. 26012567 10.1182/blood-2015-01-620583 PMC4536890

[34] M. A. Zahoor , G. Xue , H. Sato , Y. Aida , Virus Res. 2015, 208, 156. 26116899 10.1016/j.virusres.2015.06.017

[35] Q. Niu , Q. Zhou , Y. Liu , H. Jiang , Sci. Rep. 2017, 7, 9075. 28831064 10.1038/s41598-017-08699-z PMC5567260

[36] J. Li , M. Zaslavsky , Y. Su , J. Guo , M. J. Sikora , V. Van Unen , A. Christophersen , S.‐H. Chiou , L. Chen , J. Li , X. Ji , J. Wilhelmy , A. M. Mcsween , B. A. Palanski , V. V. A. Mallajosyula , N. A. Bracey , G. K. R. Dhondalay , K. Bhamidipati , J. Pai , L. B. Kipp , J. E. Dunn , S. L. Hauser , J. R. Oksenberg , A. T. Satpathy , W. H. Robinson , C. L. Dekker , L. M. Steinmetz , C. Khosla , P. J. Utz , L. M. Sollid , et al., Science 2022, 376, eabi9591. 35258337 10.1126/science.abi9591 PMC8995031

[37] A. Barade , S. Santhakumar , B. George , E. S. Edison , Blood. 2018, 132, 3872.

[38] U. Dirksen , K. A. Moghadam , C. Mambetova , C. Esser , M. Führer , S. Burdach , Pediatr. Res. 2004, 55, 466. 14681495 10.1203/01.PDR.0000111201.56182.FE

[39] J. Zhao , Y. Jia , D. Mahmut , A. A. Deik , S. Jeanfavre , C. B. Clish , V. G. Sankaran , Cell. 2023, 186, 732. 36803603 10.1016/j.cell.2023.01.020 PMC9978939

[40] K. Schnurr , A. Borchert , H. Kuhn , FASEB J. 1999, 13, 143. 9872939 10.1096/fasebj.13.1.143

[41] S. Jin , C. F. Guerrero‐Juarez , L. Zhang , I. Chang , R. Ramos , C.‐H. Kuan , P. Myung , M. V. Plikus , Q. Nie , Nat. Commun. 2021, 12, 1088. 33597522 10.1038/s41467-021-21246-9 PMC7889871

[42] G. Yu , L.‐G. Wang , Y. Han , Q.‐Y. He , OMICS. 2012, 16, 284. 22455463 10.1089/omi.2011.0118 PMC3339379

[43] K. Gennady , S. Vladimir , B. Nikolay , S. Boris , N. A. Maxim , S. Alexey , bioRxiv 2021, 060012, 10.1101/060012.

[44] Y. Hao , S. Hao , E. Andersen‐Nissen , W. M. Mauck , S. Zheng , A. Butler , M. J. Lee , A. J. Wilk , C. Darby , M. Zager , P. Hoffman , M. Stoeckius , E. Papalexi , E. P. Mimitou , J. Jain , A. Srivastava , T. Stuart , L. M. Fleming , B. Yeung , A. J. Rogers , J. M. Mcelrath , C. A. Blish , R. Gottardo , P. Smibert , R. Satija , Cell. 2021, 184, 3573. 34062119 10.1016/j.cell.2021.04.048 PMC8238499

[45] D. Zhang , Y. Li , C. Du , L. Sang , L. Liu , Y. Li , F. Wang , W. Fan , P. Tang , S. Zhang , D. Chen , Y. Wang , X. Wang , X. Xie , Z. Jiang , Y. Song , R. Guo , J. Transl. Med. 2022, 20, 363. 35962439 10.1186/s12967-022-03566-6 PMC9373312

[46] N. Zhou , X. Yuan , Q. Du , Z. Zhang , X. Shi , J. Bao , Y. Ning , L. Peng , Nucleic Acids Res. 2022, 51, D571. 10.1093/nar/gkac935 PMC9825716 36305834

