# Supplementary material for: Unbiased Single‐Cell Sequencing of Hematopoietic and Immune Cells from Aplastic Anemia Reveals the Contributors of Hematopoiesis Failure and Dysfunctional Immune Regulation
Source: Adv Sci (Weinh). 2023 Dec 25;11(10):2304539. doi: 10.1002/advs.202304539 (PMC10933602; doi:10.1002/advs.202304539)

ADVANCED SCIENCE
Open Access

Supporting Information

for *Adv. Sci.*, DOI 10.1002/advs.202304539

Unbiased Single-Cell Sequencing of Hematopoietic and Immune Cells from Aplastic Anemia Reveals the Contributors of Hematopoiesis Failure and Dysfunctional Immune Regulation

Rongqun Guo, Jingjing Kong, Ping Tang, Shuya Wang, Lina Sang, Liu Liu, Rong Guo, Ketai Yan, Mochu Qi, Zhilei Bian, Yongping Song*, Zhongxing Jiang* and Yingmei Li**

Twins with aplastic anemia: A Case Report

Abstract

INTRODUCTION

Aplastic anemia (AA) is a rare disorder characterized by bone marrow failure mediated by hyperactivated T cells. The causes of AA are varied. To shed light on the origins of AA, we discussed the outcomes of hematopoietic cell transplantation in genetically-identical twins affected by AA. Hereby, we present a case report of twins with AA and their unique treatment strategy.

CASE PRESENTATION

A 30 years old male (Twin #1, 2022), recently diagnosed with aplastic anemia, presented with symptoms of pancytopenia and cutaneous hemorrhaging.

Many years ago, the father of the individual died from acute myeloid leukemia. In the year 2017, Twin #1, acting as a donor, supplied hematopoietic stem cells to his identical twin sibling (Twin #2) who was diagnosed with AA. The individual underwent a comprehensive diagnostic process, which included routine blood tests, bone marrow biopsy, blood iron, transferrin and ferritin tests, total iron binding capacity (TIBC), immunophenotyping, and other essential examinations. Peripheral blood counts demonstrated an absolute neutrophil count (ANC) of 780 cells/μl, a platelet count of 8K/μl, hemoglobin of 145g/L, and an absolute reticulocyte count of 27K/μl. The lymphocyte ratio exhibited an elevated proportion, while the presence of megakaryocytes and platelets in the bone marrow was scarce. The hematopoietic island exhibited a vacant grid-like arrangement, consisting of granulocytes, lymphocytes, and a limited quantity of erythrocytes. The results of H&E and PAS staining revealed a significantly reduced level of hematopoietic tissue proliferation in the bone marrow (less than 10%), accompanied by a greater presence of bone tissue and an increased amount of adipose tissue. The percentage of $CD20^{+}$ B cells is 2.8%, indicating a decrease compared to the standard range. Additionally, the presence of $CD34^{+}CD117^{+}$ early myeloid cells and $CD10^{+}CD19^{+}$ B precursors is infrequent. Chromosome analysis revealed no aberrations. Twin #1 initiated a treatment regimen involving the use of TPO and TPO-RA in conjunction with G-CSF. Twin #2 expressed a strong desire to serve as a donor for hematopoietic

stem cells to support his younger sibling. Following a comprehensive assessment, Twin #2 has been deemed a suitable and healthy candidate for providing hematopoietic stem cells. Currently, Twin #1 is exhibiting satisfactory progress in recovery, while Twin #2 maintains a state of good health. Further details can be found in Figure 7.

Supplemental Material

Supplemental Table Legends

Supplemental Table 1. Characteristics of healthy donors, and patients with AA or PNH.

Supplemental Table 2. Details of mass cytometry files.

Supplemental Table 3. Details of cell number of each sample and each subset, and proportion of each subset.

Supplemental Table 4. Supplemental Table 4. Gene sets of ferroptosis suppressor and driver

Supplemental Table 5. TH-related gene sets.

Supplemental Table 6. δ1 and δ2 T features.

Supplemental Table 7. CytoATLAS mass cytometry antibodies from Polaris Biology

Supplemental Table 8. Characteristics of healthy donors and patients with AA using for cytokine detection.

Supplemental Material 1. Twins with aplastic anemia: A Case Report

Supplemental Figure Legends

Supplemental Figure 1.

(A) Dot plot showing the relative expression and proportion of cell type-relevant markers in each subpopulation. Each cluster was annotated based on the expression status of the differentiation markers.

(B) Proportions of each subpopulation in each sample from HDs and patients with AA.

(C) Circle plots showing the number (i) and strength (ii) of interactions in all merged BM- and PB-derived cell types. The round loops along with the cell type, represent interactions within the same cell type. (iii) Chord diagram showing all significant interactions (L-R pairs) between different cell types.
(D) Representative flow plots showing the surface markers in AA and HD groups.
(E) The BM plasma concentrations of multiple chemokines (CCL3, CCL11, CXCL8, CXCL10, CCL5, and CCL4) and cytokines (bFGF, IL1RA, IL-2, IL-5, IL-7, IL-10, IL-12, IL-15, IL-17, VEGF, TNF-α, INF-γ, IL-9, and PDGF-BB) were measured using high-sensitivity multiplex cytokine assays. *P* value were determined using an unpaired two-tailed Student's t test: $*P < 0.05$, $**P < 0.01$, $***P < 0.001$, and $****P < 0.0001$.

Supplemental Figure 2. Hyperactivated $CD8^+$ T cells contribute to the autoimmune response and HSPC destruction.

(A) Dot plot showing genes specifically expressed in the major $CD8^+$ T cell types. Red represents high expression, and cyan-blue represents low expression. The circle represent the percentage of cells expressing the indicated genes.
(B) Representative flow cytometry plots of KIR^+CD8^+ T cells in PBMCs and BMMCs from patients with AA and HDs.
(C) Dot plot showing the effector-related genes of the major $CD8^+$ T cell types.
(D) The percentage of $CD45RA^+CCR7^+$ naïve $CD8^+$ T cells was measured using MC (i) and flow cytometry (ii).
(E) Dot plot showing the *TNFSF10* expression in $CD8^+$ T cells from different groups.
(F) Dot plot showing the *TNFSF10* expression in major $CD8^+$ T cell types.
(G) Dot plot showing TNFSF10 receptor genes (*TNFRSF10A*, *TNFRSF10B*, *TNFRSF10C*, and *TNFRSF10D*) in HSPCs from different groups.
(H) Proportion of $CXCR4^+$ cells in the BM and PB $CD8^+$ T cells of patients with AA and HDs (HDPB, n=5; AAPB, n=11; HDBM, n=3; AABM, n=11). *P* value was determined using an unpaired two-tailed Student's t-test: $**P <$ 0.01.
(I) Violin plot shows the expression of *CD69* in $CD8^+$ T cells from different groups.
(J) MC analysis of BM HSPCs from HDs and patients with AA.

Supplemental Figure 3.

(A) UMAP display of scRNA-seq data from $CD4^+$ T cells from HDs and patients with AA.
(B) Dot plot showing the expression of selected markers in different $CD4^+$ T cell subsets.
(C) Violin plots (i) and dot plots (ii) showing the expression of selected genes in $CD4^+$ Tconv cells from HDs and patients with AA. $*P < 0.05$, $**P < 0.01$, $***P < 0.001$, $****P < 0.0001$.
(D) Experimental schema.
(E) UMAP visualization of scRNA-seq data from Tregs of HDs and patients with AA.
(F) Dot plot showing expression of selected genes in different Treg subsets.
(G) The GSEA enrichment analysis score identified resting/activated Tregs. The selected gene sets were "GSE15659_RESTING_VS_ACTIVATED_TREG_DN" and "GSE15659_RESTING_VS_ACTIVATED_TREG_UP."
(H) Violin plot showing expression of selected genes in Tregs.
(I) Several gene sets for cell death (WP_TNFRELATED_WEAK_INDUCER_OF_APOPTOSIS_TWEAK_SIGNALING_PATHWAY, BIOCARTA_TNFR1_PATHWAY, REACTOME_APOPTOSIS, HAMI_APOPTOSIS_VIA_TRAIL_UP, WP_FERROPTOSIS, REACTOME_PYROPTOSIS) to assess the sensitivity of different subsets to cell death.
(J) Percentages of $CCR4^-FAS^-$, $CCR4^+$, and FAS^+ subsets in Tregs.

Supplemental Figure 4. Characteristics of pathogenic γδ T subpopulation.

(A) UMAP plot of representative expression patterns of *CD247*, *NCAM1*, *KLRB1*, *FCGR3A*, and *CD3E*.
(B) UMAP visualization of scRNA-seq data from NK, NKT, and γδ T cells of HDs and patients with AA (i). The bar plot shows the proportion of NK, NKT, and γδ T cells in each sample (ii).
(C) Distribution of $TCRv\delta2^+$ T subset in γδ T cells in PB and BM samples from patients with AA and HDs.
(D) Representative dot plots showed the CD45RA and CD27 expression of BM γδ T cells.
(E) Representative histogram overlay showing expression of CD27, CD16, CD160, and HLA-DR by BM γδ T cells from HDs or patients with AA. Dot plots exhibited *KLRK1* expression in γδ T cells, NKT cells, and NK cells of HDs and patients with AA.
(F) Representative dot plots showing the gating strategy for the identification of different NK or NKT cell subsets

based on CD56 and CD16 expression.

Supplemental Figure 5. scRNA-seq analysis of B lineages, erythroid progenitors, and myeloid cells showed contributors to an autoimmune response.

(A) Dot plot showing the expression of selected genes per B lineage subset identified in Figure 5A.

(B) Percentages of apoptotic and dead $CD19^+$ B cells in patients with AA and HDs. Fresh (< 2 h) PBMC were collected from five patients with AA and four HDs.

(C) Dot plot showing the expression of immune-related effectors in different subsets.

(D) Representative histogram overlay showing HLA-DR expression in BM $CD19^+/CD20^+$ B cells from HD or AA samples.

(E) Dot plot illustrating the discriminant gene sets for each erythroid progenitor stage.

(F) The bar plot shows the proportions of the four erythroid stages in each sample.

(G) Circle plots showing the number (i) and strength (ii) of interactions in NK/T and erythroid cells. Round loops, along with the cell type, represent interactions within the same cell type.

(H) Chord diagram showing selected L-R pairs between different cell types and concrete contributions of each L-R pair.

(I) Representative signaling pathways (BAG6-NCR3-PS and NECTIN1-CD96) from the erythroid S1 and erythroid S2.

(J) UMAP embedding of myeloid cells (DC, monocytes, macrophages, and neutrophils) and pDC from integrated datasets colored according to cell type.

(K) Proportion of bars in the individual groups.

(L) Violin plot shows the expression of *TNFSF10* in the different groups.

(M) Important ligand-receptor pairs from myeloid cells to other immune cells (i). The dotted plot shows the expression levels of these receptors and ligands in the different subsets and groups (ii).

Supplemental Figure 6. The ferroptosis status of HSCs and lineage-restricted progenitors.

(A) Ferritin concentration in patients with AA.

(B) UMAP plot colored according to the ferroptosis suppressor score (i). The violin plot shows the ferroptosis suppressor scores (ii).

(C) UMAP plot colored according to the ferroptosis driver score (i). Violin plot showing the ferroptosis driver scores (ii).

(D) The dot plot shows normalized, scaled expression (color) and the proportion of non-zero-expressing cells (circle size) of marker genes per cluster and annotated cell types.

(E) UMAP plot showing the expression levels of *ACSL4*, *ALOX15*, *ALOX5*, *LPCAT3*, *ALOX12*, and *SAT1* in HSPCs.

(F) Expression levels of ferroptosis-related genes in each subpopulation of the different samples.

(G) Three-dimensional (3D) PCA of BM plasma samples based on the contribution of oxidized fatty acids.

(H) Representative plots showing ROS levels in PB $CD34^+$ HSPCs and $CD3^+$ T cells from HDs and patients with AA.

Supplemental Figure 7. MC analysis of twin P3.

Supplemental Figure 8. Venn diagram of upregulated/downregulated genes in different cell types of patients with AA. The differentially expressed genes were got by performing *"FindMarkers(object, ident.1= ident.1, ident.2= ident.2, method="DESeq2")*" function in each cell type of BM cell in patients with AA and HDs. "*ident.1*" was defined as AA group, and "*ident.2*" was defined as HD group. These genes were grouped into upregulated or downregulated. The *VennDiagram* package was used to identify intersections.

Supplemental Figure 9. The expression of *MYSM1* in HSPCs.

(A) Dot plot showing the expression levels of *MYSM1* in different groups.

(B) Dot plot showing the expression levels of *MYSM1* in different HSPC subpopulations.

Supplemental Figure 10. Schematic of mechanisms that induce AA.

Supplemental Figure 1

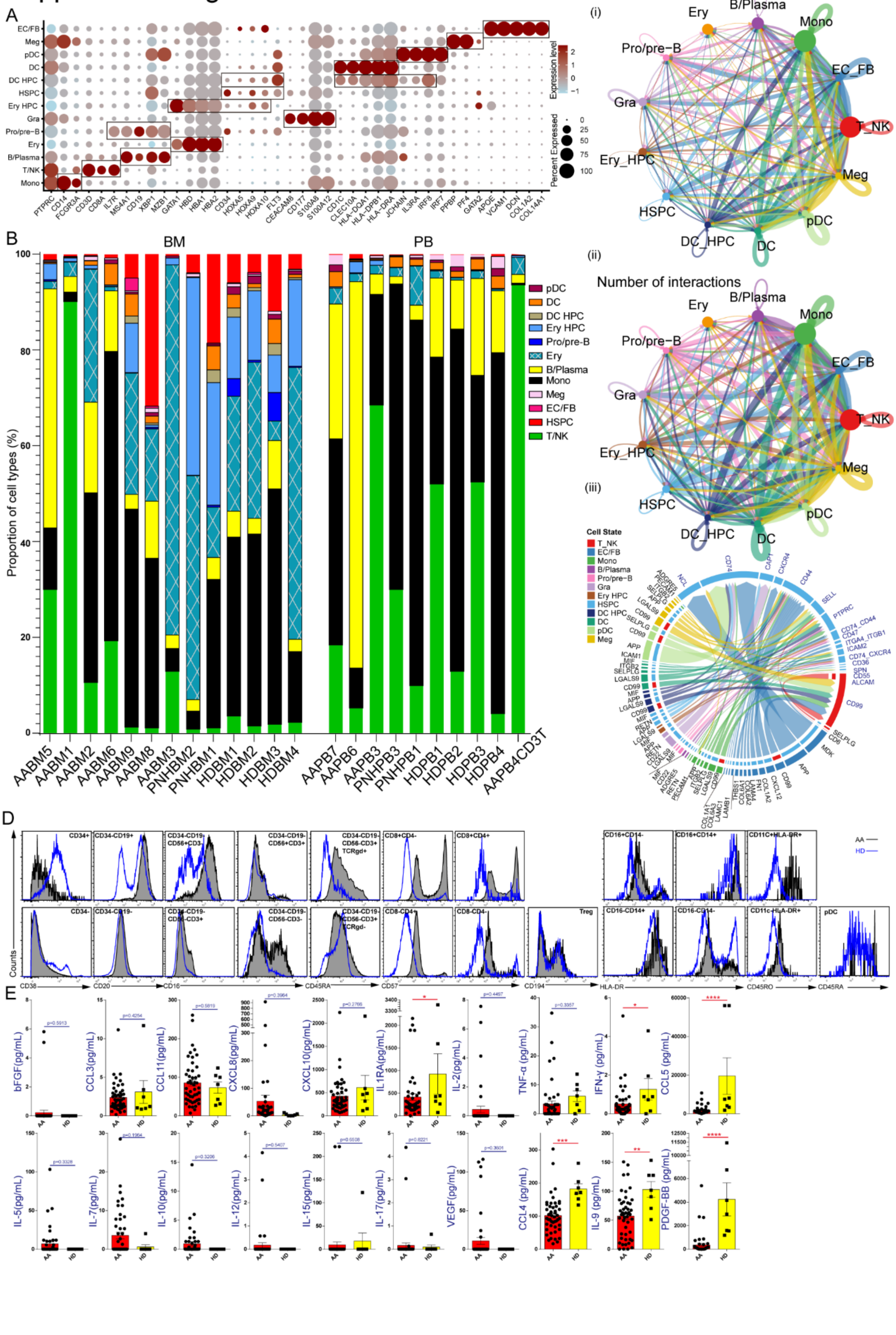

Supplemental Figure 2

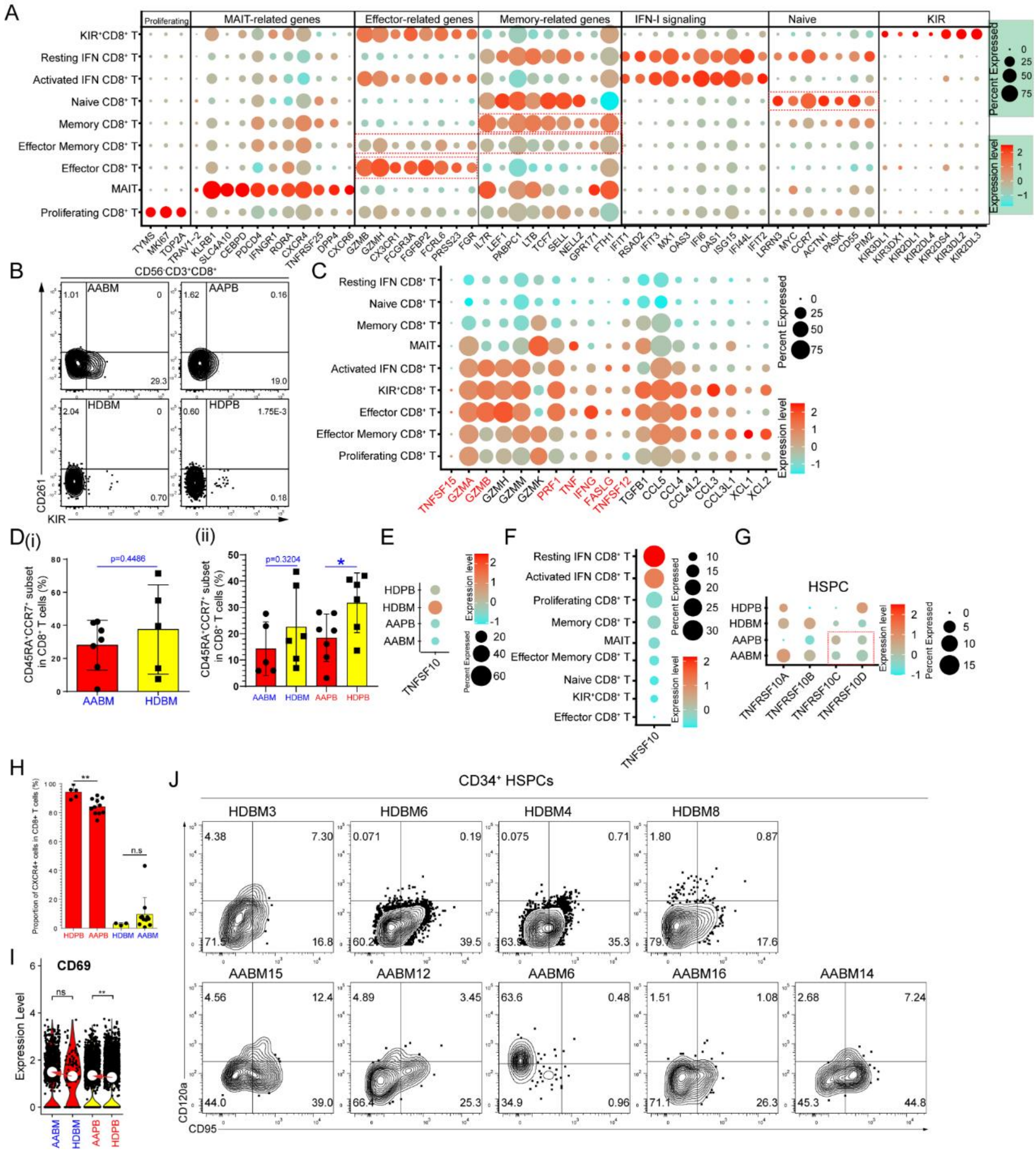

Supplemental Figure 3

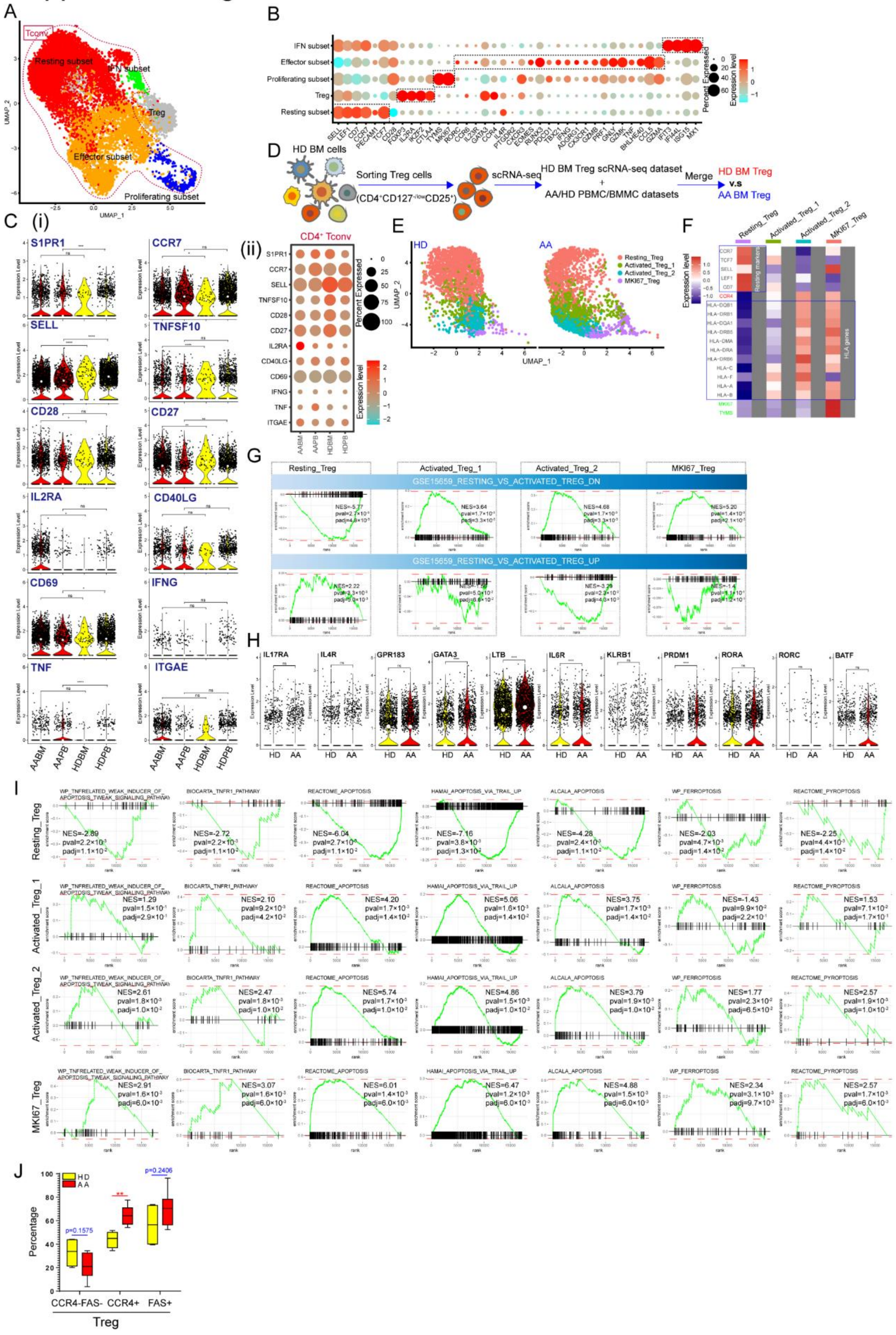

Supplemental Figure 4

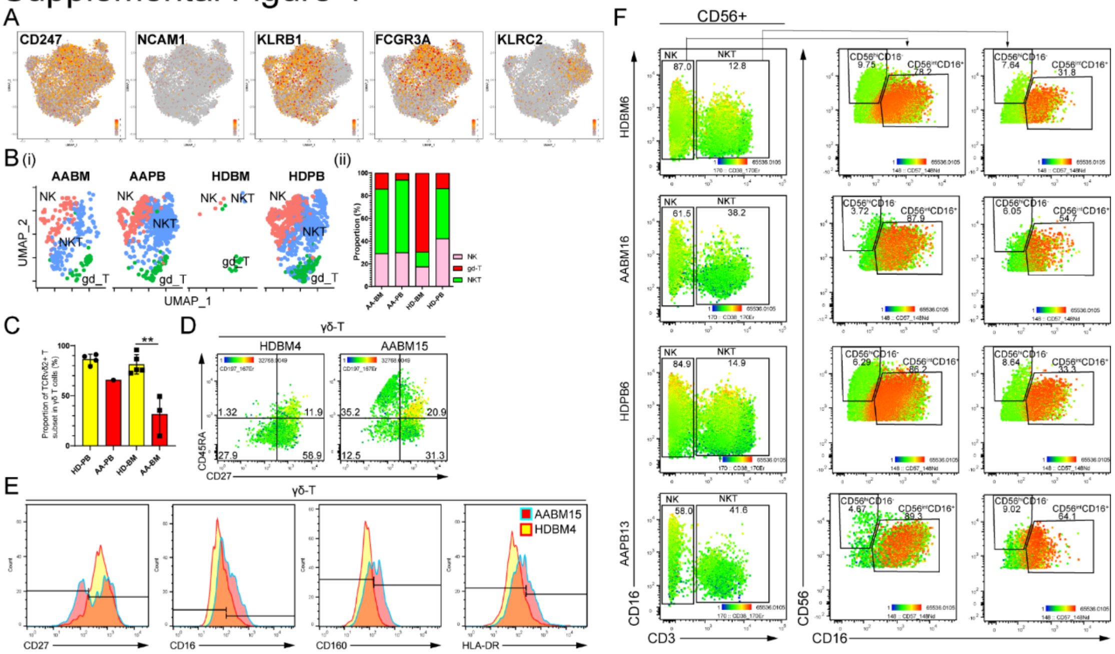

Supplemental Figure 5

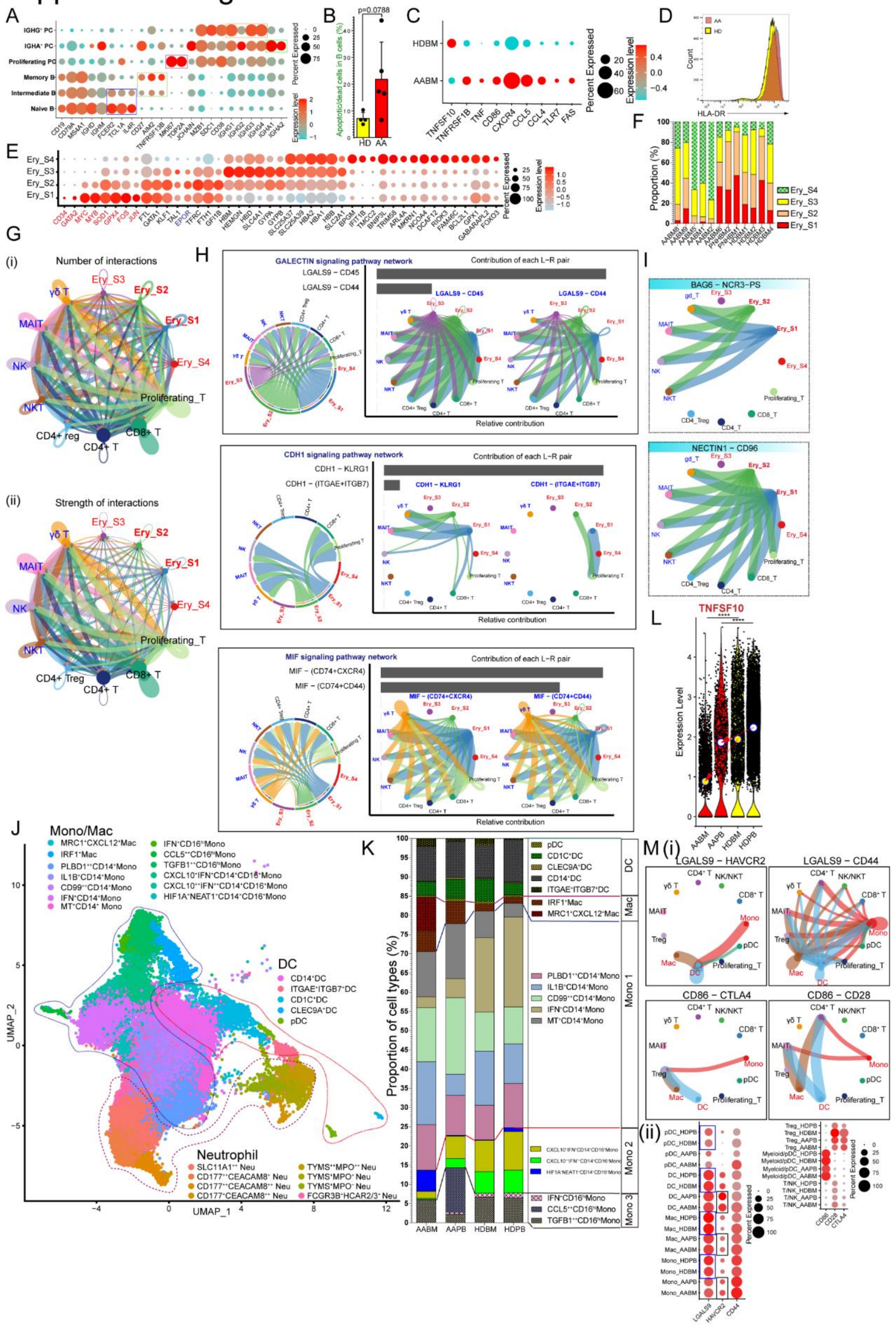

Supplemental Figure 6

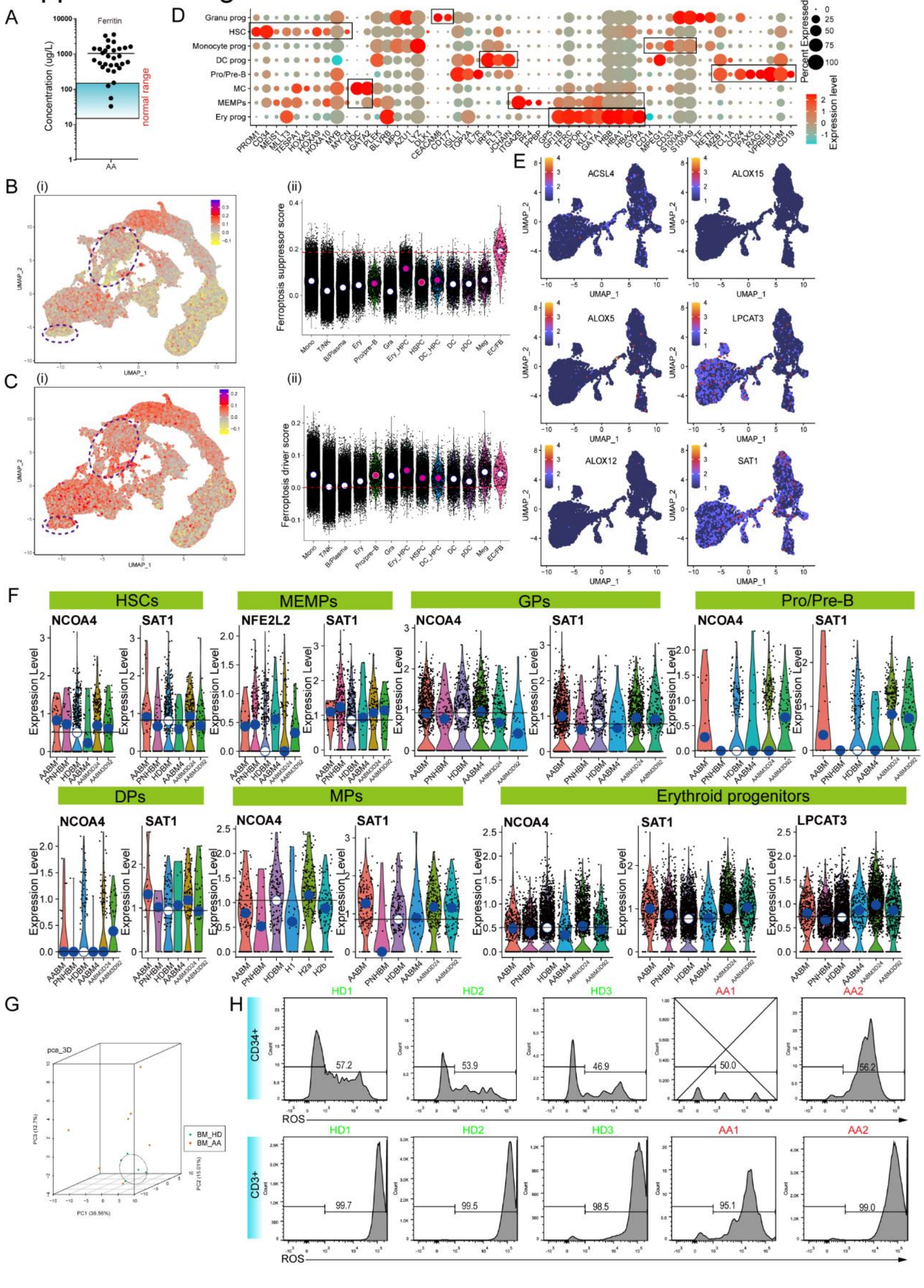

Supplemental Figure 7

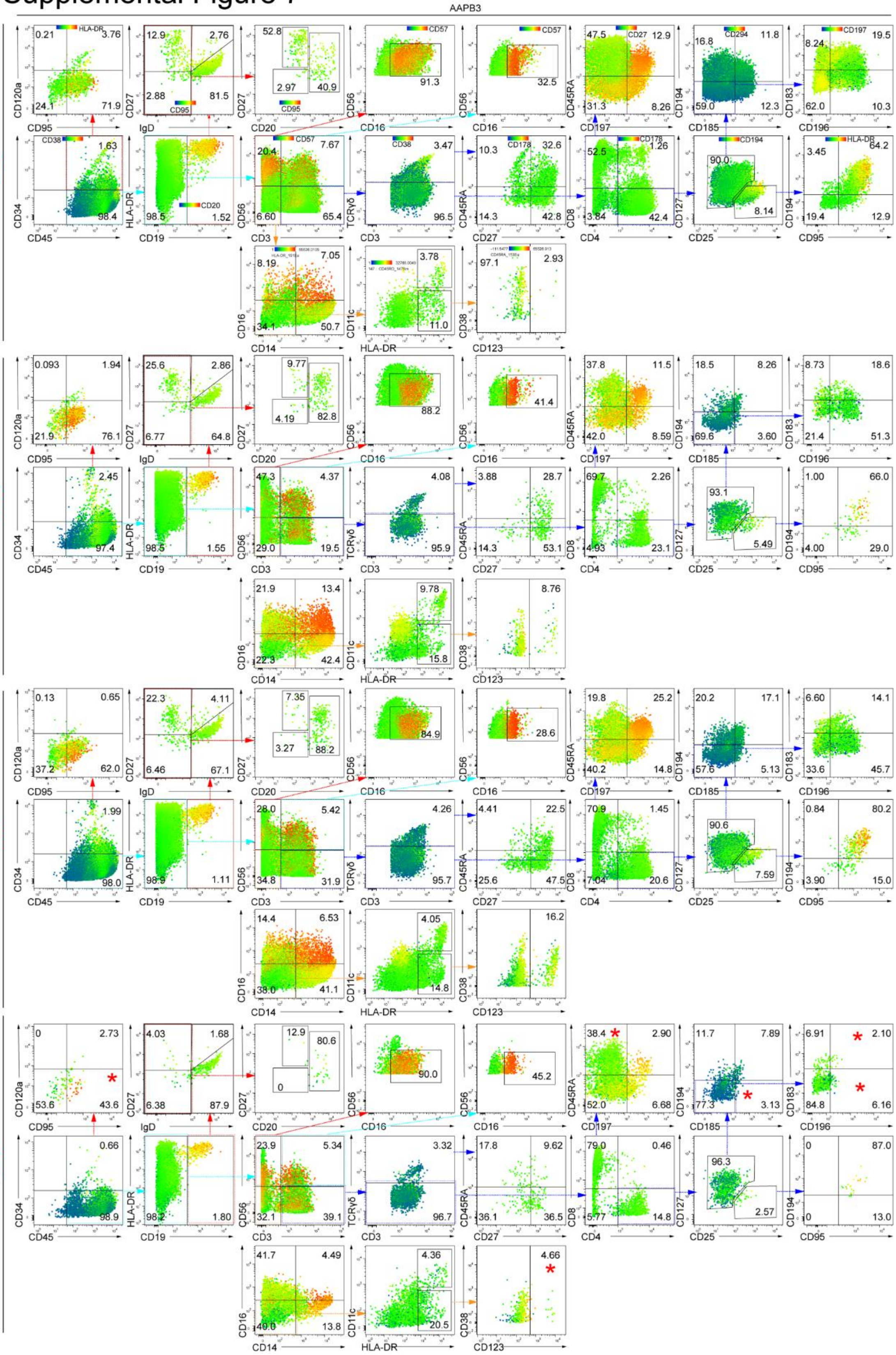

Supplemental Figure 8

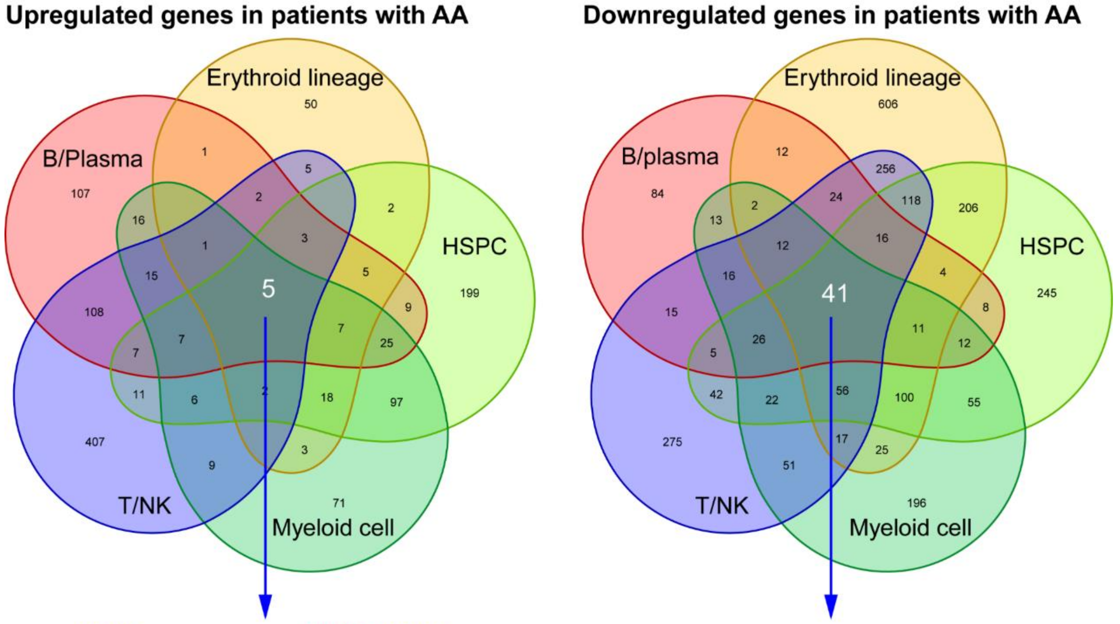

MT2A, CCL5, NKG7, IFITM2, MT1E

HMGN3, TMEM14B, APEX1, BANF1, DNMT1, PSMA4, CACYBP, TIMM13, HSP90AA1, HIST1H1C, NME4, NDUFAF3, SIVA1, MGST2, IFI44, BEST1, DNAJA1, UBE2L6, EIF1AY, RNF130, PRDX2, HSPA8, BLVRB, RPS4Y1, SOX4, DYNLL1, IFIT3, H1FX, IFI44L, BST2, XAF1, IFI6, CA1, MX1, JUN, TUBA1B, STMN1, AHSP, ISG15...

Supplemental Figure 9

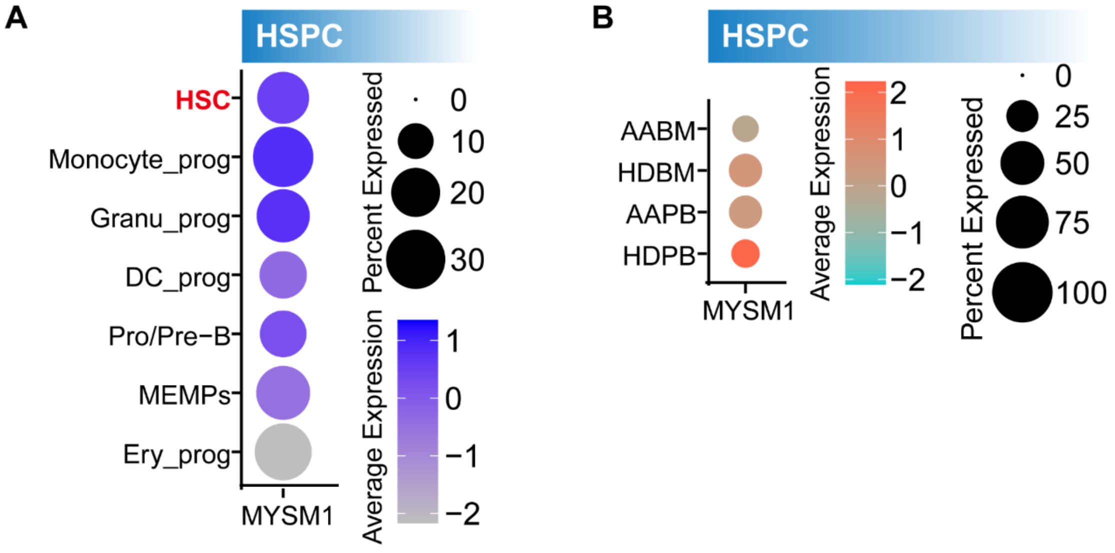

Supplemental Figure 10

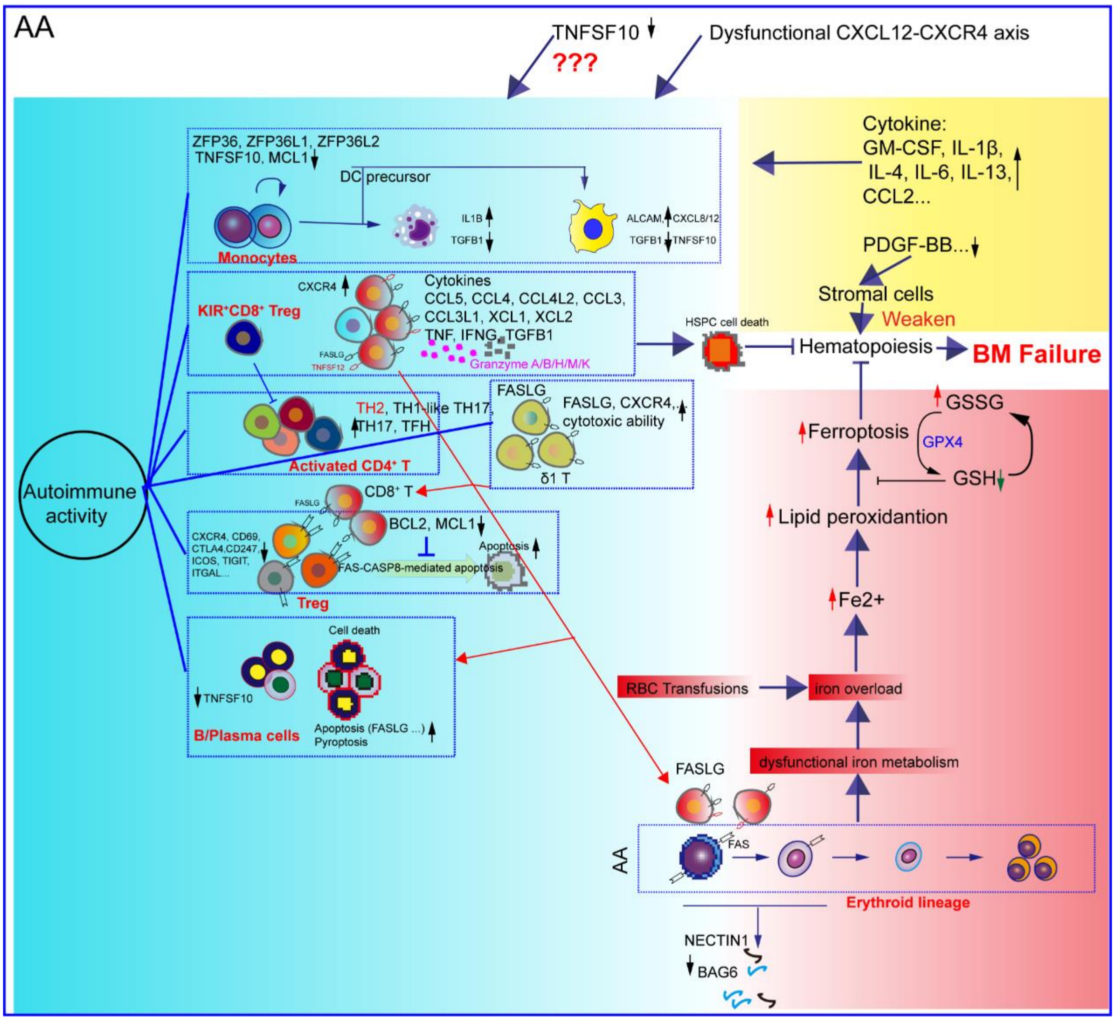

Supplement: Supplementary file 1 — Supporting Information [file ADVS-11-2304539-s002.pdf]
